# Intracrystalline deformation microstructures in natural olivine with implications for stress estimation

**DOI:** 10.1038/s41598-022-24538-2

**Published:** 2022-11-22

**Authors:** Jian Ma, Wenlong Liu, Yi Cao, Junfeng Zhang, Chuanzhou Liu

**Affiliations:** 1 State Key Laboratory of Geological Processes and Mineral Resources, School of Earth Sciences, China University of Geosciences, Wuhan, 430074 China; 2 State Key Laboratory of Lithospheric Evolution, Institute of Geology and Geophysics, Chinese Academy of Sciences, Beijing, 100029 China; 3 CAS Center for Excellence in Tibetan Plateau Earth Sciences, Beijing, 100101 China; 4 University of Chinese Academy of Sciences, Beijing, 100049 China

**Keywords:** Structural geology, Geophysics, Tectonics, Mineralogy

## Abstract

Constraining the stress related to lithospheric deformation in natural rocks is key to develop and test a geodynamic model. However, the cautions of extrapolating piezometers that are established on experimental samples to natural rocks are less addressed. In this study, we investigated the microstructures of a natural harzburgite sample using the electron backscatter diffraction (EBSD) technique. Subgrain boundary (SGB) geometries suggest large percentages of (010)[100] and {0kl}[100] dislocation slip systems in olivines. More importantly, multiple low-angle misorientation boundaries (LAMBs) variants are recognized for the first time in olivine based on their distinctive characteristics with the change of EBSD mapping step size. The LAMBs that exist at a small step size (≤ 1 μm) are mostly equivalent to real SGBs, while other LAMBs that appear only when the step size is larger (> 1 μm) are artificial SGBs. Besides, the former develop mainly in the high LAMB density grains, whereas the latter are mostly found in the low LAMB density grains. This result reinforces the previous knowledge that the stress calculated using subgrain-related piezometers is meaningful only when real SGBs are captured at sufficiently small step size. Furthermore, we provide a proof of concept that SGB density and kernel average misorientation (KAM) are two viable metrics to estimate stress. These two alternative piezometers, which still need calibrations using the experimentally deformed samples, are anticipated to have wide applications in natural rocks.

## Introduction

A vital part of geodynamics research is to quantitatively determine the tectonic stress that caused the past and present deformation in the crust and lithospheric mantle^1–4^. The presence of experimentally calibrated correlations between the stress and deformation microstructures (i.e., piezometers), suggests that tectonic stress conditions in the deformed rocks can be inferred from the latter. Hitherto, various piezometers have been proposed, among which three are most frequently used.

The first is the dislocation density piezometer. It is based on the relation between the density of a line defect (i.e., dislocation) and the stress in minerals such as olivine^5–8^ and quartz^6^. A higher dislocation density implies a larger stress. The second is the dynamically recrystallized grain size piezometer, which is based on an inverse empirical relation between stress and the grain size of dynamically recrystallized minerals in a mono-phase aggregate, such as olivine^1,9–11^, calcite^12^, hematite^13^, orthopyroxene^14,15^, and quartz^16–18^. The third is the subgrain-size piezometer, which is built on the empirical relation between the spacing (i.e., subgrain size) of adjacent low-angle boundaries (i.e., subgrain boundary (SGB) or dislocation wall) in the minerals and stress. This piezometer has also been calibrated for olivine^6,10,11,19–21^, quartz^19,22^ and calcite^12,23^. Similarly, there is also an inverse correlation between subgrain size and stress.

The deformation microstructures (i.e., dislocation density, dynamically recrystallized grain, and subgrain size) with relevance to the above piezometers can be measured by direct observations under optical microscope e.g.^7,10–12^, scanning electron microscope e.g.^24^, and transmission electron microscope e.g.^7,9,25,26^. Besides, they can also be calculated indirectly via high-angular resolution electron backscatter diffraction (EBSD)^27^ and/or traditional EBSD mapping^13,14,16,19^.

In this study, we investigated the deformation microstructures, especially the characteristics of low-angle misorientation boundaries (LAMBs) in olivine, of a natural harzburgite sample collected from the Yarlung Zangbo suture zone in the Tibetan Plateau (the sample location is shown in Fig. A1 and see Supplementary Text for more detailed geological background and sample information). Based on these results, we clarified the influence of EBSD mapping parameters, especially step size, on the observed LAMB features and its implications for stress estimations, and proposed other potential metrics to estimate stress and their cautions when applying to natural samples.

**Figure 1:**
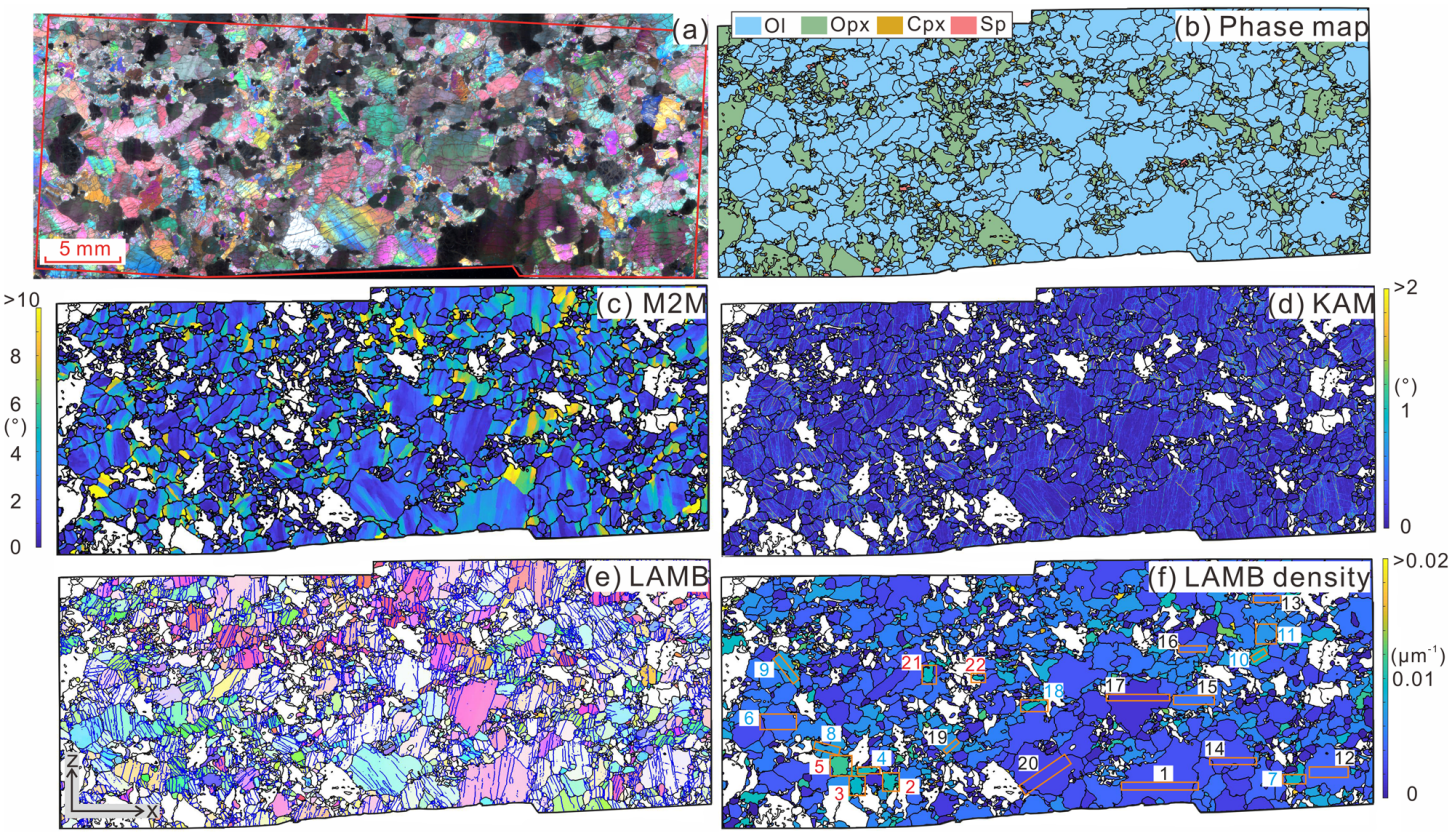
Microstructures of the studied harzburgite sample. (**a**) Cross-polarized image showing a porphyroclastic texture and extensive undulose extinctions and subgrains in olivine. (**b**) EBSD phase map showing the modal composition. (**c**) M2M map showing intracrystalline lattice distortions in olivine. Distributions of (**d**) KAM, (**e**) LAMBs (blue lines) and (**f**) LAMB density (ρ_LAMB_, see Results Section for its definition) are shown for olivine grains. EBSD data were collected using the step size of 15 μm. In (**f**), the numbered boxes denote the analyzed domains of the 22 olivine grains, and colors of the text correspond to the three groups of olivine classified by LAMB density.

## Results

### Microstructures

The studied sample 13LQ117 shows a fresh harzburgitic composition characterized by mainly olivine (~ 78 vol.%) and orthopyroxene (~ 20 vol.%), minor clinopyroxene (~ 1.3 vol.%) and spinel (~ 0.2 vol.%), and a trace amount of amphibole (Fig. 1a, b; Supplementary Table S1). It displays a porphyroclastic texture, in which large olivine and orthopyroxene grains coexist with their fine-grained counterparts (Fig. 1a, b), with mean grain sizes of olivine and orthopyroxene at about 1.5 and 1 mm, respectively (Supplementary Fig. S1a, b; Table S1). The olivine long axes are aligned predominantly subparallel to the foliation and lineation (Supplementary Fig. S2a). Olivine shows distinct intracrystalline plasticity features characterized by widespread undulose extinction and subgrain boundaries (Fig. 1a). The abrupt color variations in the mis-to-mean (M2M, i.e., misorientation angle between each pixel and their mean orientation of a grain) map (Fig. 1c) and the bright streaks in the kernel average misorientation (KAM, i.e., average misorientation angle between each pixel and its nearest neighbors within the grain, depending on the choice of kernel size) map (Fig. 1d) match well with the locations of low-angle misorientation boundaries (LAMBs, see Results Section for its definition) (Fig. 1e). The unevenly distributed M2M, grain orientation spread (GOS, i.e., arithmetic mean of M2M in a grain), KAM and LAMBs reflect heterogeneous and extensive intracrystalline lattice distortions in olivines (Figs. 1c–f and Fig. A2 in the Supplementary Text). Olivine grains have straight to slightly curved boundaries and are weakly elongated (Fig. 1a, Supplementary Figs. S1c, e and Table S1). Notably, the orientations of LAMBs tend to be normal to the foliation (Fig. 1e and Supplementary Fig. S2d).

**Figure 2:**
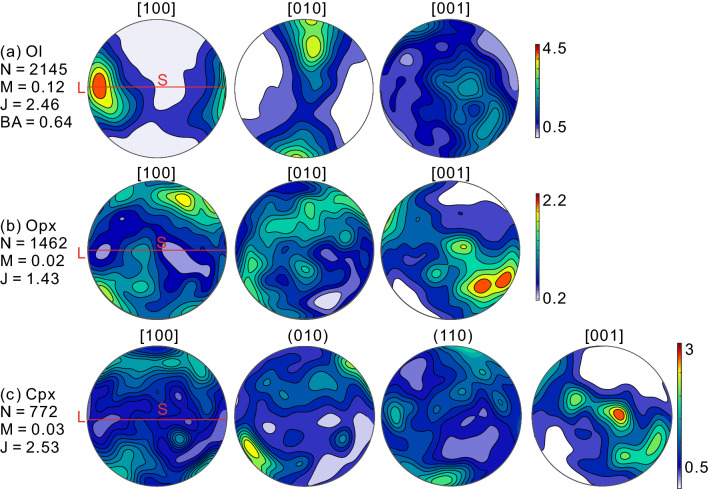
One-point-per-grain crystallographic preferred orientations of (**a**) olivine, (**b**) orthopyroxene and (**c**) clinopyroxene in the studied samples. N: number of grains, M: M-index, J: J-index, and BA: BA-index. L and S are the lineation and foliation, respectively.

Similar to olivine, orthopyroxene grains have curvilinear boundaries and show some undulose extinction and LAMBs (Fig. 1a). Compared with olivine, orthopyroxene grains are smaller in size and have larger shape factor and aspect ratio (Supplementary Fig. S1b, d, f; Table S1). Besides, M2M, GOS, KAM and LAMB density of orthopyroxene are overall lower than those of olivine (Supplementary Fig. S3). Clinopyroxene lamellae can be observed in orthopyroxene, and vice versa. Clinopyroxene grains in the matrix have an irregular shape, a small mean grain size (~ 170 μm), and rarely develop LAMBs and undulose extinction. Orthopyroxene grains lack a discernable shape preferred orientation (Supplementary Fig. S2b), whereas spinel grains are well aligned, marking the foliation and lineation (Supplementary Fig. S2c) that are consistent with those defined by the olivine grains.

### Crystallographic preferred orientations

In sample 13LQ117, olivine shows a relatively strong CPO (J-index ~ 2.46 and M-index ~ 0.12) characterized by a high concentration of [100] axes subparallel to the lineation, the girdle-like distribution of the [010] axes with a subordinate concentration sub-perpendicular to the foliation (Fig. 2a). This CPO can be classified as A/D-type, which is intermediate between A- and D-type CPOs, see also^28,29^, and indicated by a high BA-index of 0.64. In comparison, both orthopyroxene (J-index ~ 1.43 and M-index ~ 0.02) and clinopyroxene (J-index ~ 2.53 and M-index ~ 0.03) display weaker and more dispersed CPOs. For both orthopyroxene and clinopyroxene, the maxima of [001] and [100] axes tend to align subparallel to the lineation and subnormal to the foliation, respectively (Fig. 2b, c), which are overall correlated with the olivine CPO, i.e., correlations of $\left[ {001} \right]_{{{\text{Opx}}}} \parallel \left[ {100} \right]_{{{\text{Ol}}}}$ and $\left[ {100} \right]_{{{\text{Opx}}}} \parallel \left[ {010} \right]_{{{\text{Ol}}}}$ with some clear obliquities (Fig. 2).

### Characteristics of olivine low-angle misorientation boundaries

#### Variations of LAMBs with step size

Distinct from subgrain boundary (SGB), which specifically indicates that a sharp lattice curvature in a discrete plane occurs over a short-range wavelength (e.g., < 1 μm in olivine) caused by dense stacking of dislocation arrays^30–34^, a low-angle misorientation boundary (LAMB) is defined here as the low-angle boundary whose misorientation angle is greater than 1° and less than 10°, derived purely from EBSD data regardless of mapping step size. In other words, the descriptive term LAMB has no meaning on the physical origins of the observed low-angle boundaries. Otherwise, the unambiguous term SGB is used in the text where its physical meaning is specially indicated. In fact, the use of a larger EBSD mapping step size may actually result in artificial SGBs (see Discussion). The density of LAMB (ρ_LAMB_) in a grain is defined as the total length of LAMBs divided by the analyzed area representing the olivine in the field of view, using the unit μm^−1^.

For olivine, LAMBs can be observed in both large and small grains in the studied sample and they are mostly straight and align sub-perpendicular to the foliation and lineation (Fig. 1e and Supplementary Fig. S2d). Based on the density of LAMBs (ρ_LAMB_) at the step size of 1 μm and its slope with step size at 1–5 μm, the 22 analyzed olivine grains can be divided into low-ρ_LAMB_ (0.169–0.891 × 10^−2^ μm^−1^), medium-ρ_LAMB_ (1.27–1.91 × 10^−2^ μm^−1^), and high-ρ_LAMB_ (2.12–2.66 × 10^−2^ μm^−1^) groups (Supplementary Table S2). In general, low-ρ_LAMB_ grains are mainly the large grains, whereas the medium- and high-ρ_LAMB_ grains are much smaller in size (Fig. 1f). The features of LAMBs in three representative grains, grain-1, grain-4 and grain-2, from each group above are shown below.

**Figure 3:**
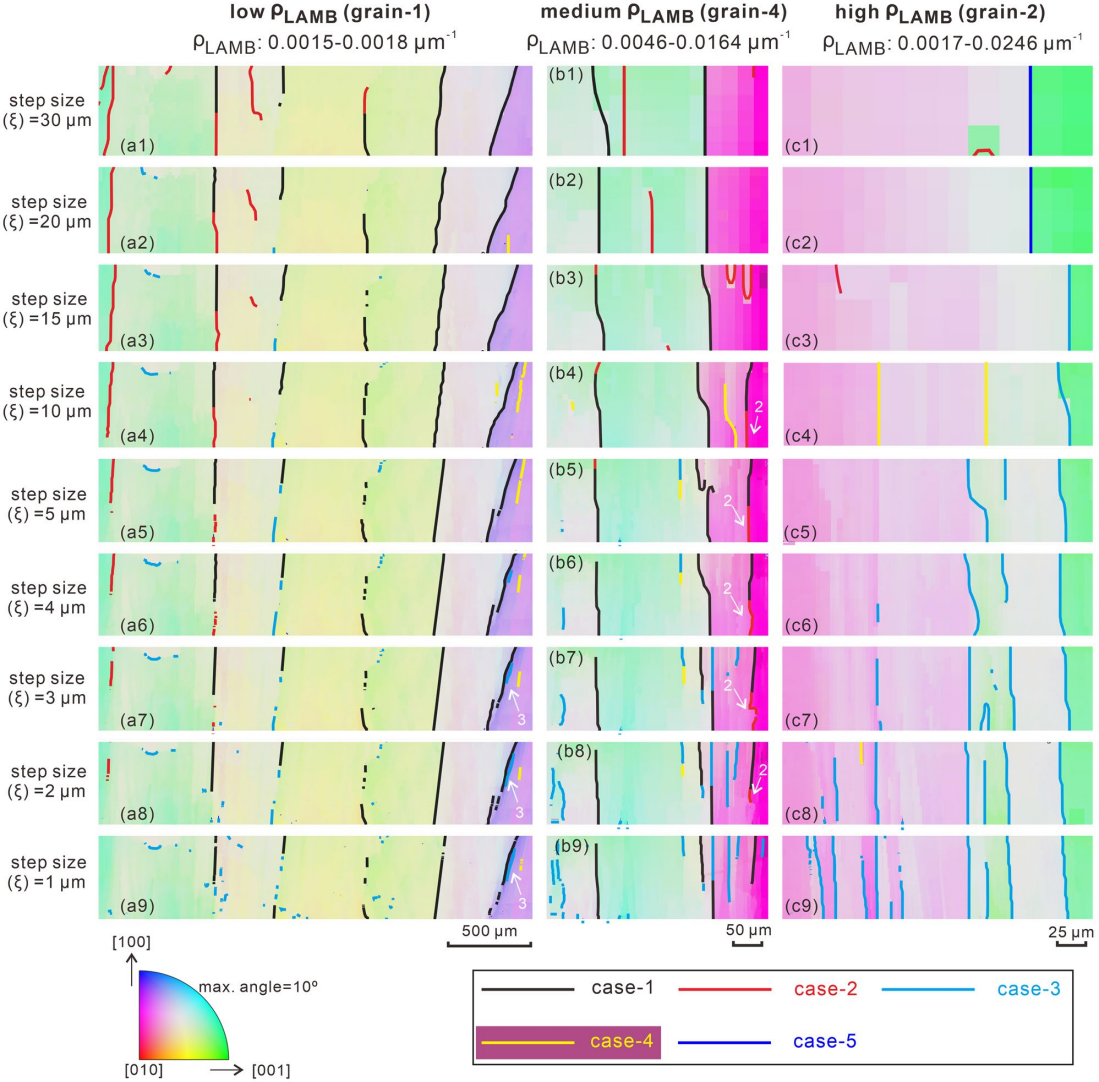
Five variants (case-1 to -5) of LAMB in three representative (**a1**–**a9**) low, (**b1**–**b9**) medium and (**c1**–**c9**) high LAMB density (ρ_LAMB_) olivine grains. All grains are color-coded with their orientations. Cavities and cracks can cause a minor amount of spurious LAMBs (irregular dots and lines), which become more striking at smaller step sizes, due to indexing errors.

Accompanied with the decrease of step size from 30 to 1 μm, at least five variants of LAMBs can be observed (Fig. 3). Case-1 LAMBs are persistent, as they can exist at all step sizes. Case-2 LAMBs exist only at larger step sizes but fade away gradually with decreasing step size. Oppositely, case-3 LAMBs emerge only at smaller step sizes but disappear gradually with increasing step size. Case-4 LAMBs are more complicated as they occur only at medium step sizes and vanish at both larger and smaller step sizes. The four LAMB variants above have relative stable locations on the map when step size changes. In contrast, some LAMBs (case-5) disappear and seem to “split” into two or more LAMBs with decreasing step size. It appears that the LAMBs in the high- and medium-ρ_LAMB_ grains are dominated by case-1 and -3 (Fig. 3b, c), while more case-2 LAMBs appear in the low-ρ_LAMB_ grains (Fig. 3a). The variation of LAMB density with step size also provides clues on how these LAMBs vary. The low-ρ_LAMB_ grains show a nearly constant LAMB density (0.0015–0.0018 μm^−1^, Fig. 3a), which implies a nearly balanced disappearance and appearance rate of different LAMB variants with step size. Conversely, LAMB densities in the medium-ρ_LAMB_ (0.0046–0.0164 μm^−1^, Fig. 3b) and high-ρ_LAMB_ (0.0017–0.0246 μm^−1^, Fig. 3c) grains vary remarkably with step size, suggesting imbalanced evolutions of different LAMB variants.

### Dislocation slip systems inferred from SGBs

**Figure 4:**
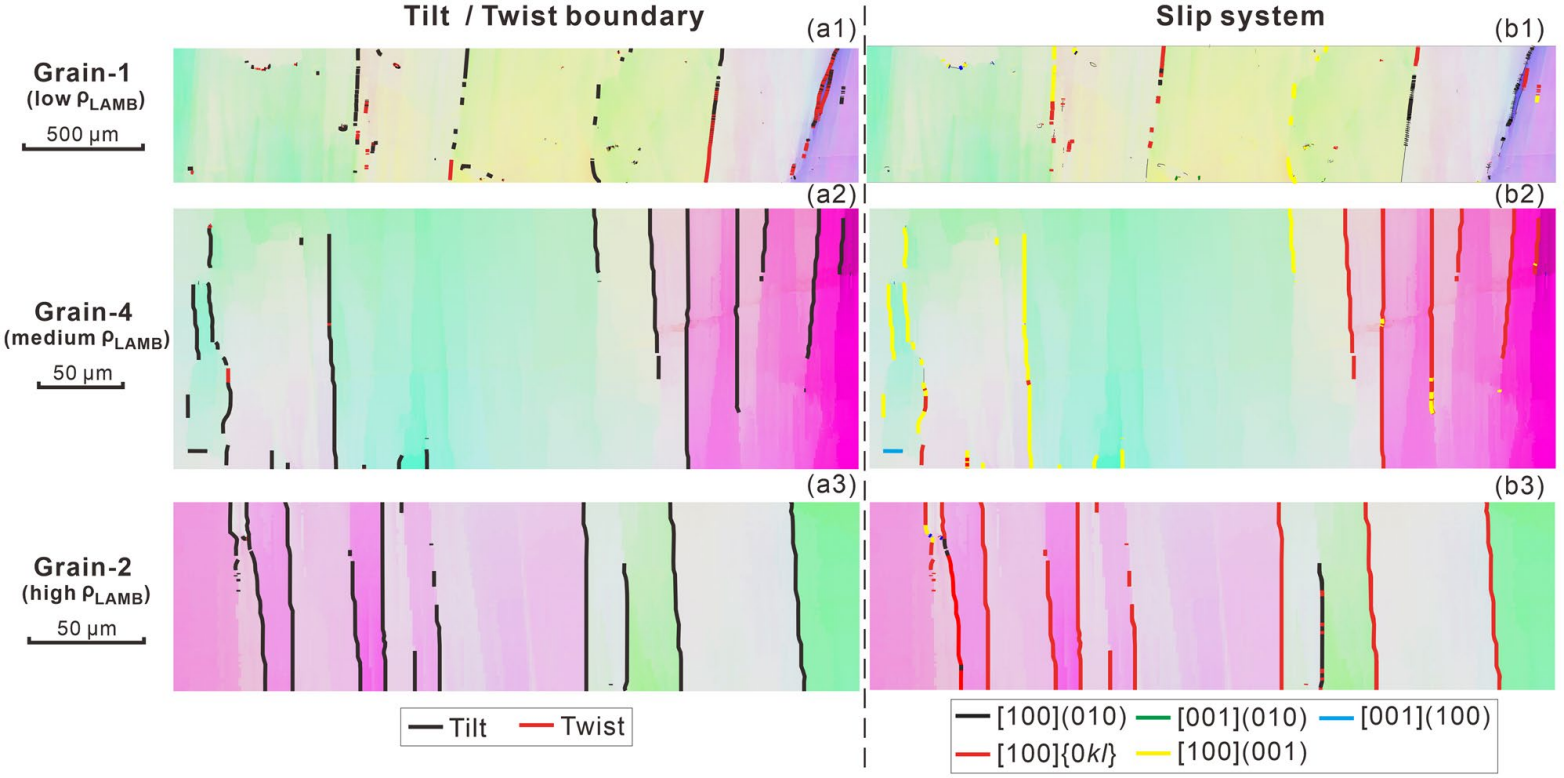
Distributions of the (**a1**–**a3**) tilt and twist SGBs and the (**b1**–**b3**) inferred dislocation slip systems for three representative low-, medium- and high-ρ_LAMB_ olivine grains, same as those in Fig. 3. Results are derived from the step size of 1 μm.

**Figure 5:**
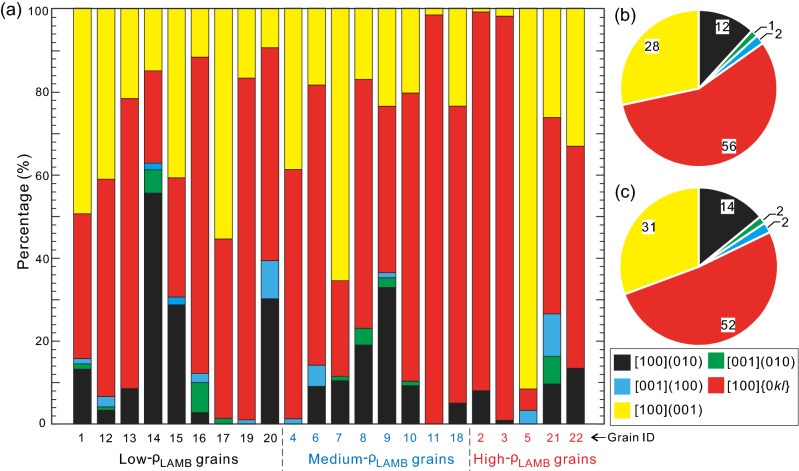
(**a**) Percentages of 5 dislocation slip systems in the 22 analyzed olivine grains. The average proportions of five slip systems are calculated in two ways: (**b**) first calculating the proportions of SGBs related to each of the 5 slip systems in each grain, then averaging them, and (**c**) first summing the length of SGBs related to each of the 5 slip systems in all grains, then calculating their averages. Results are derived from the step size of 1 μm.

As shown in the Discussion below, LAMB is equivalent to SGB when the step size is small enough. Therefore, we assume that the LAMBs recovered at the minimum step size of 1 μm in this study are predominantly SGBs, from which different dislocation slip systems can be inferred. According to the geometrical relation between the SGB trace and the axis of rotation, SGB can be divided into tilt (rotation axis aligning subparallel to SGB trace) and twist (rotation axis aligning subperpendicular to the SGB trace) boundaries. The analyses of SGBs retrieved at the step size of 1 μm in all 22 olivine grains show that SGBs are dominantly tilt boundaries, as their percentages are 60.3–99.9%, 49.8–95.9% and 41.1–88.6% for the low-, medium- and high-ρ_LAMB_ grains, respectively. Overall, the percentages of tilt boundary are over 80% for most grains (Fig. 4a; Supplementary Table S2). The SGB length proportions of edge dislocation slip systems that form the tilt boundaries vary greatly among the all 22 grains, i.e., (010)[100] ~ 0–55.7%, (010)[001] ~ 0–6.7%, (100)[001] ~ 0–10.2%, {0kl}[100] ~ 5.1–98.3%, (001)[100] ~ 0.9–91.6% (Fig. 5a; Supplementary Table S2). When taken together, {0kl}[100], (001)[100] and (010)[100] are the majorities, as their average percentages are 52–56%, 28–31% and 12–14%, respectively (Fig. 5b, c; Supplementary Table S2). Likewise, no obvious correlation between the dislocation slip systems and LAMB density is observed.

### Relations between KAM, LAMB density, intercept length, and step size

**Figure 6:**
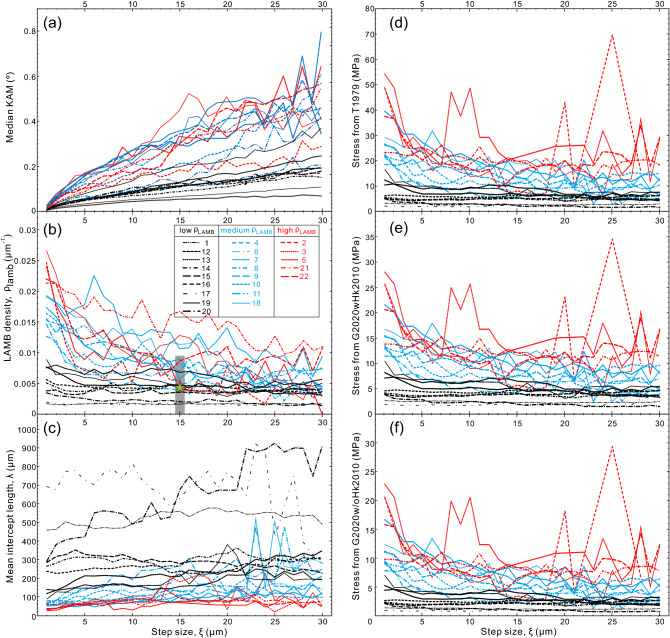
Variations of (**a**) median KAM, (**b**) LAMB density, (**c**) intercept length, and (**d**–**f**) stress with step sizes from 1 to 30 μm in the 22 analyzed olivine grains, which are classified into low-, medium- and high-ρ_LAMB_ grains. For the large-area EBSD mapping data using the step size of 15 μm, the grey bar is the range of arithmetic mean plus and minus one standard deviation, and the green star is area-weighted average. Some grains (e.g., grain-2 and grain-3) yield null values of mean intercept length for some step sizes, because they show only one (or zero) LAMB and these data are excluded as outliers. Stress is estimated using subgrain-size piezometers provided by (**d**) T1979, (**e**) G2020wHK2010, and (**f**) G2020w/oHK2010.

Overall the median KAM increases with step size and varies in the ranges of 0.007°–0.37°, 0.009°–0.80° and 0.014°–0.65° in low-, medium- and high-ρ_LAMB_ grains, respectively (Fig. 6a; Supplementary Table S2). The KAMs in low-ρ_LAMB_ grains are markedly lower than those in the medium- and high-ρ_LAMB_ grains, and the latter two show largely overlapped KAM ranges, together implying a moderate correlation between KAM and LAMB density. Besides, KAM fluctuates considerably at step sizes larger than 10 μm in the medium- and high-ρ_LAMB_ grains (Fig. 6a).

The LAMB density fluctuates weakly and increases slightly with step size in the low-ρ_LAMB_ grains. However, in the medium- and high-ρ_LAMB_ grains, LAMB density shows striking fluctuation and increment with decreasing step size, which is especially prominent in high-ρ_LAMB_ grains (Figs. 3 and 6b). For comparison, the LAMB density varies in a large range of from 0 to 9.9 × 10^−3^ μm^−1^, with the area-weighted mean of 4.37 × 10^−3^ μm^−1^ in large-area mapping data (grey area and green star in Fig. 6b). This range of LAMB is mostly overlapped with that of the 22 olivine grains produced at the step size of 15 μm. The area-weighted mean LAMB density is similar to the LAMB density of low-ρ_LAMB_ grains, owing to the large area of such grains.

At the step size of 1 μm, the mean intercept length ranges from 88 to 692 μm in the low-ρ_LAMB_ grains, whereas smaller mean intercept lengths of 37–79 μm and 27–63 μm are found in the medium- and high-ρ_LAMB_ grains, respectively (Fig. 6c; Supplementary Table S2). This correlation is readily comprehensible, because higher LAMB density is equivalent to a narrower spacing between neighboring LAMBs. The intercept length generally decreases with the reducing step size, and it fluctuates dramatically at large step sizes in some grains, especially the high- and medium-ρ_LAMB_ grains, due to their small grain sizes (Fig. 6c).

### Stress

At the same step sizes, the stresses calculated by T1979 are larger than those estimated by G2020wHK2010 and G2020w/oHK2010 (Fig. 6d–f; Supplementary Table S2). For the 22 olivine grains and at the step size of 1 μm, the average stresses (mean ± one standard deviation) by T1979, G2020wHK2010, and G2020w/oHK2010 are 22 ± 15.4, 12.8 ± 7.7 and 9.3 ± 6.5 MPa, respectively. Stress displays a trend of decrease with increasing step size, which is especially striking at the small step sizes (Fig. 6d–f). The fluctuations of stress with step size are also opposite to those of intercept length (cf. Fig. 6c–f). Because of the higher LAMB density and smaller intercept length, stresses of the high-ρ_LAMB_ grains are considerably larger than those of the medium- and low-ρ_LAMB_ grains (Fig. 6d–f). This phenomenon is more pronounced at the step size of 1 μm, where the stresses estimated by T1979 are 7.4 ± 4.5, 25.7 ± 6.1 and 42.5 ± 11 MPa for the low-, medium- and high-ρ_LAMB_ grains, respectively. This difference weakens at the step size of 15 μm (Fig. 6d). The same patterns are found in the stresses estimated by G2020wHK2010 and G2020w/oHK2010 (Fig. 6e, f).

## Discussion

### Deformation mechanism

The porphyroclastic microstructure, undulose extinction, well-developed SGBs, and distinct CPOs clearly suggest that deformation is mainly accommodated by dislocation slip. The presence of an olivine A/D-type CPO is indicative of the activation of dominant (010)[100] and {0kl}[100] dislocation slip systems^35^, which is overall consistent with the dislocation slip systems inferred from SGBs (Figs. 4 and 5). However, the prevalence of the (001)[100] slip system (28–31%) over (010)[100] (12–14%), which was also recently reported by Lopez-Sanchez et al.^34^, is contradictory to the observation of an A-type CPO component. The reason for this discrepancy may be related to faster or more extensive recovery of the SGBs consisting mainly of the (010)[100] slip system compared to the SGBs composed majorly of the (001)[100] slip system on the geological time scale, thus more former SGBs than the latter ones evolve into grain boundaries^28^. Alternatively, the proportion of the different dislocation types in the SGBs needs not be in direct proportion to the activity of the different slip systems that accommodate the overall deformation. The reason is that (010)[100] dislocations can move unimpededly through the crystal without leaving any (or hardly any) misorientation gradients or dislocation walls (i.e., SGBs)^34,36^, or that grain-to-grain interaction can activate the hard-slip dislocations and thus vary the proportion of different dislocation types in SGBs locally^34^. In these cases, the presence of the (001)[100] slip system related SGBs does not contradict with the dominant (010)[100] and {0kl}[100] slip systems in the deformation process. This discrepancy has also been recently observed in the experimentally deformed samples^34,37^.

The alignments of [100]_Ol_, [001]_Opx_ and [001]_Cpx_ crystallographic axes subparallel to the lineation indicate coeval deformation of olivine and two pyroxenes. However, both orthopyroxene and clinopyroxene are characterized by relatively dispersed CPOs and weaker strength (J-index ~ 2.69 and 2.47) and an oblique angle between their [001]-axis maxima and the olivine [100]-axis maximum (Fig. 2). This oblique angle and dispersed CPO of pyroxenes are commonly observed in naturally deformed peridotites e.g.^28,38–40^ and are usually attributed to the lower finite strains accommodated by stronger pyroxenes compared with weaker olivine crystals^41,42^. The orthopyroxene CPO shows maxima of the [001] and [100] axes aligning subparallel to the lineation and subnormal to the foliation, respectively (Fig. 2b, also named as Type-AC CPO by Jung, et al.^43^. This CPO type of orthopyroxene has also been reported in many previous natural peridotite studies e.g.^28,40,44,45^, indicating that the deformation of orthopyroxene is mainly accommodated by the (100)[001] dislocation slip system^46^.

### Genesis of various LAMBs and their relations to SGB

**Figure 7:**
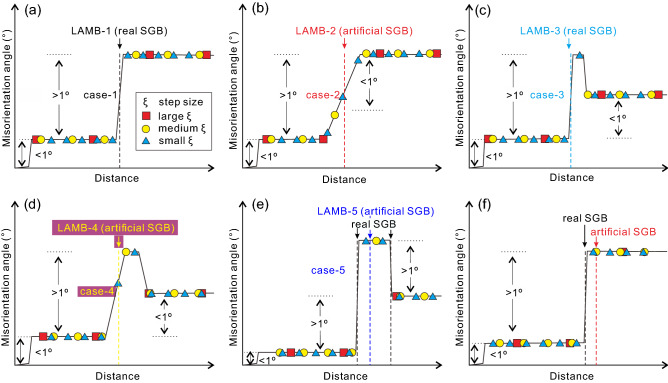
Schematic diagrams explaining the formations of five observed LAMBs (**a**–**e**) and another case (**f**) with the variation of EBSD mapping step size. A misorientation threshold of 1° between adjacent pixels is used to define LAMB (colored vertical dashed lines) here.

In this study, we define LAMBs artificially in terms of the above-threshold misorientation angles between adjacent pixels that are derived from EBSD mapping data. Therefore, the existence and morphology of LAMBs are closely related to the choices of the threshold value, mapping position, and step size, and the gradient of lattice orientation in a grain. As described above, at least five variants of LAMBs are recognized based on their distinctive evolutions with step size (Fig. 3). The feasible origins of different LAMBs are envisioned as below. As step size decreases gradually from large to small values, the misorientation angle between adjacent pixels is always greater than the threshold defining a LAMB (e.g., 1° in this study) due to a large misorientation gradient, hence LAMB could always exist (i.e., case-1 LAMB, Fig. 7a). If there exists a region with a large misorientation gradient and above-threshold misorientation, but the misorientation angle is lower than the threshold at larger step sizes, then LAMB appears only when neighboring pixels straddle just across the large misorientation gradient region when the step size is smaller (i.e., case-3 LAMB, Fig. 7c). This case has also been reported when measuring grain size and subgrain size in previous literature^16,19,47^. Because case-1 and -3 LAMBs occur at a small step size, which suggests the existence of a high lattice curvature, these two variants of LAMBs are primarily composed of real SGBs^30–32^. On the contrary, when the misorientation gradient is relatively low, the misorientation angle between adjacent pixels decreases continuously with the insertion of pixels at smaller step sizes, leading to a gradual disappearance of LAMB (i.e., case-2 LAMB, Fig. 7b). This variant of LAMB agrees with the definition of a deformation band, which has a gradual lattice curvature over a long-range wavelength and can be considered as a transitional texture from undulose extinction to SGB during progressive recovery^31^. Therefore, a case-2 LAMB is actually not an SGB. For the case-4 LAMB, a low misorientation gradient hampers the appearance of an LAMB at the smaller step sizes and a below-threshold misorientation inhibits the appearance of an LAMB at the larger step sizes, while an LAMB only emerges when above-threshold misorientation is achieved at medium step sizes (Fig. 7d). Since the case-4 LAMB has no sharp lattice curvature, this LAMB variant is not a real SGB either. The case-5 LAMB is difficult to interpret. Over a long distance, multiple real SGBs exist. However, only one LAMB appears when the step size is large and its location differs obviously from those of SGBs (Fig. 7e), indicating that this variant of LAMB is also an artificial SGB. Dependent on the mapping position, a real SGB can shift its location at large step size, creating artificial SGBs (Fig. 7f).

In sum, an LAMB is equivalent to an SGB when the EBSD mapping step size is small enough (case-1 and -3), while it develops into various spurious SGBs at larger step sizes (case-2, -4, -5 and more). Besides, it is noted that the manner that lattice curvature manifested in EBSD maps is essentially a continuum effect, in that there is an endless range of possible distributions and magnitudes of lattice curvature, which can be sampled by a continuous range of step size. This fact will generate a wide range of observational effects including and likely going beyond the five variants of LAMB observed in this study. The LAMB patterns documented here are explicit and their categorization could be helpful to illustrate the key differences among the various LAMBs discovered in this work. However, they should not be regarded simply as a discretization of the full continuum effects as mentioned above.

The LAMB variants observed in this study appear to correlate with the LAMB density in the grains (see Section of *Variations of LAMBs with step size*). The LAMBs are mainly case-1 and case-3 (real SGBs) in the high- and medium-ρ_LAMB_ grains, whereas case-2 LAMBs (artificial SGBs) are more prevalent in the low-ρ_LAMB_ grains (Fig. 3). At the early stage of deformation or lower strain and stress, dislocation density is low as indicated by a sparse distribution of dislocations, the number or density of LAMBs is therefore limited. The dislocations are loosely stacked in the vicinity of an LAMB, resulting in a low misorientation gradient and case-2 LAMB represented deformation bands. As deformation (i.e., strain) advances and stress increases, the dislocation density increases correspondingly, and dislocations slip and climb closer to form a narrow dislocation wall. This process stacks the previous loose dislocations on the LAMBs more densely and produces a larger misorientation gradient, forming case-1 and -3 LAMBs represented SGBs and a high LAMB or SGB density.

### Implications for stress estimation

#### Can KAM be used to estimate stress?

Kernel average misorientation (KAM), which is usually derived from the EBSD mapping data, is an important metric to measure the local lattice misorientation^48^. It currently has been widely employed to analyze the intracrystalline deformation microstructures in minerals e.g.^49^. KAM is thought to result mainly from the geometrically necessary dislocations (GNDs) that are caused by stress or plastic deformation, although inherent crystal defects such as solute atom, second phase, and grain boundary can also contribute partially to the local lattice curvature or distortion^48^. This argument is supported by the close relation between KAM and dislocation density^50,51^, suggesting that KAM, like dislocation density, can be used theoretically as a proxy to constrain stress.

**Figure 8:**
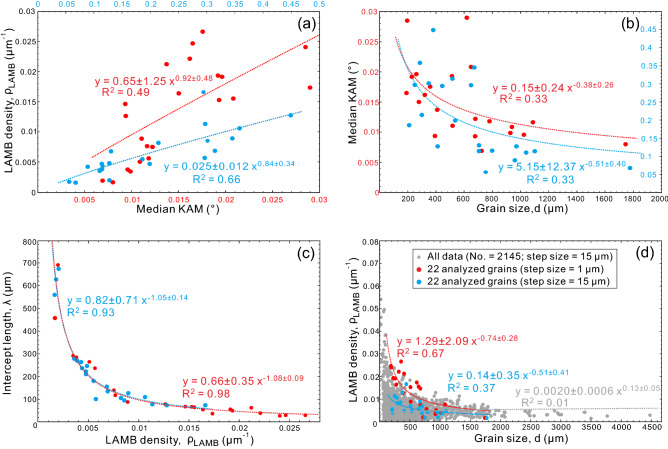
(**a**) LAMB density versus median KAM, (**b**) median KAM versus grain size, (**c**) intercept length vs. LAMB density and (**d**) LAMB density versus grain size. Since grain-3 has only one LAMB at the step size of 15 μm, its intercept length cannot be calculated and excluded in (**c**). The colors of the fitting curves and the fitting equation correspond to the colors of scatters.

**Figure 9:**
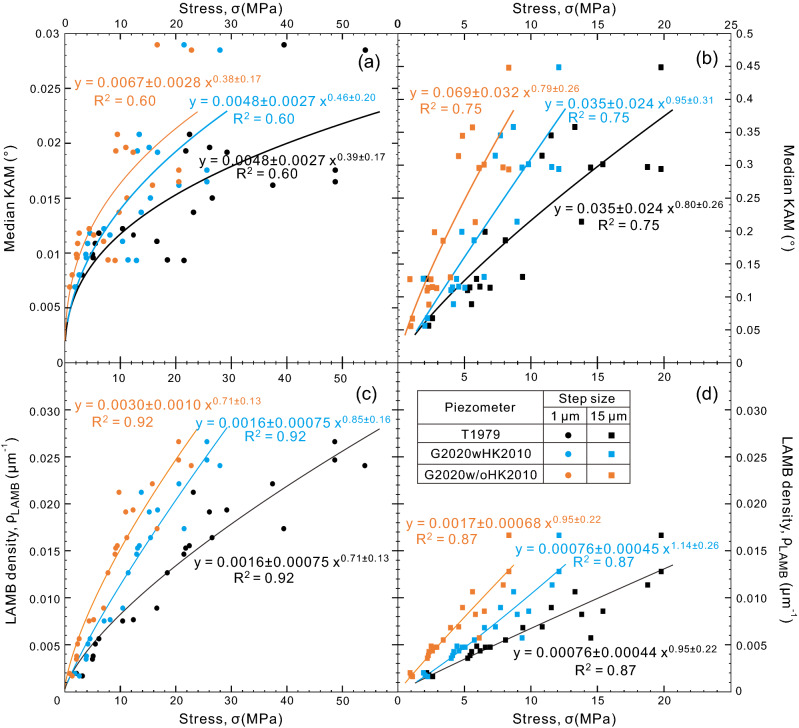
Relations between median KAM and stress (**a** and **b**) and between LAMB density and stress (**c** and **d**) for the 22 analyzed olivine grains at the step size of 1 μm (circles, left column) and 15 μm (rectangles, right column). The results of step size of 15 μm here are obtained by magnification from 1 μm (see Methods). Grain-3 has only one LAMB at the step size of 15 μm and its subgrain size cannot be calculated, this grain is thus excluded in stress estimation. Stress is estimated using the subgrain-size piezometers of T1979 (black), G2020wHK2010 (cyan) and G2020w/oHK2010 (orange).

In our sample, high KAM values correspond well with the locations of LAMBs (Fig. 1d, e), implying that KAM may also correlate closely with the density of LAMB or SGB, as well as with stress. For step sizes of 1 and 15 μm, the correlations between KAM and LAMB or SGB density (R^2^ = 0.49 and 0.66, Fig. 8a) are moderate and slightly higher correlations between KAM and stress are observed (R^2^ = 0.60 and 0.75, Fig. 9a, b). These fair correlations may partly be attributed to the arbitrary choice of kernel size over which KAM is calculated. In contrast, KAM and grain size are weakly correlated (R^2^ = 0.33 and 0.33, Fig. 8b). Compared to grain orientation spread (GOS), which is not a viable metric to estimate stress (see Supplementary Text for more explanations), KAM has several benefits. First, KAM avoids the problem caused by averaging misorientations over a grain, which can result in a low GOS but a high LAMB or SGB density. Second, KAM is estimated over the entire area of the grain considered and there is no need to weight the result by grain area to obtain a meaningful average value. Third, KAM is also straightforward to calculate using the built-in functions in MTEX. In this case, KAM is expected to be a viable metric to estimate stress in future.

### Can SGB density be used to estimate stress?

In a grain with a fixed size, a smaller distance between the neighboring LAMBs indicates that there exist more LAMBs (i.e., higher LAMB density). Our results support this argument, as intercept length has strong negative correlations with LAMB density (R^2^ = 0.98 and 0.93, Fig. 8c). Besides, based on the stress calculated from the intercept length, we found that there are also strong positive correlations between the LAMB density and the stress (R^2^ = 0.92 and 0.87 for step sizes of 1 and 15 μm, respectively, Fig. 9c, d).

The subgrain-size piezometer implies that the smaller size of the subgrain, the greater stress it represents. In this study, subgrain size is represented by the linear intercept length between neighboring LAMBs. However, this assumption is valid only when LAMBs are SGBs (i.e., case-1 and -3 LAMBs), which occur when the step size is sufficiently small. In other words, the estimated stress is meaningful unless the density of SGBs is measured. The minimum step size of 1 μm produces predominantly case-1 and -3 LAMBs (i.e., SGBs) in the EBSD maps (Fig. 3a9, b9 and c9), implying that the intercept length approximates subgrain size, that LAMB density approaches SGB density, and that the calculated stress is most likely sound in this situation, although an even smaller step size (< 0.5 μm) is suggested to be required to capture nearly all SGBs and authentic subgrain size in the experimentally deformed olivines^19^. It is noteworthy that the value of this critical step size apparently depends on the spacing of adjacent SGBs in a grain, which needs to be at least twice the step size^19^.

The appearance of artificial SGBs (e.g., case-2, -4, -5 and more LAMBs) indicates that the subgrain size and its deduced SGB density will yield erroneous stress estimates when using a large EBSD mapping step size (e.g., 15 μm). This case is clearly exemplified by the remarkably different stresses estimated from 1 μm and 15 μm step sizes (Figs. 6 and 9; Supplementary Table S2). The reason we raised this issue is that a large step size is very often used, especially when one maps natural coarse-grained samples in a large domain. To deduce a meaningful stress value using subgrain size and its derived piezometers, we must be cautious about the step size selected and examine whether the LAMBs it produces are real SGBs or not.

A clear negative correlation between LAMB or SGB density and grain size for the step size of 1 μm (R^2^ = 0.67, Fig. 8d) indicates that the stress inferred from an LAMB or SGB density may be consistent with the stress inferred from a dynamically recrystallized grain size e.g.^9^. The observation of olivines with a smaller grain size tending to develop a higher LAMB or SGB density and smaller subgrains (Fig. 8d), implies that smaller sized olivines may be affected by greater stress. This finding may be interpreted by the fact that the smaller grains have smaller contact areas with their neighboring grains, thus concentrating a considerably high stress in the small grains^52,53^. The widespread distributions of olivine subgrain size, LAMB or SGB density, and grain size may reflect that the distribution of stress is also spatially heterogenous in the sample, which is consistent with the inhomogenous distribution of GNDs in deformed polycrystalline aggregates^52,54^. Furthermore, it is noted that the orientation of a grain to the applied stress can determine the resolved shear stress on a dislocation slip system, and thus also affect the development of LAMBs or SGBs in individual grains. In other words, the density of LAMBs or SGBs in a single grain is a combined effect of grain orientation and stress intensity. However, their relative importances are still unknown but can be quantified using deformation experiments on single crystals in future.

Compared to measuring subgrain size using intercept lengths, it is easier to obtain LAMB or SGB density, because the density of LAMB or SGB can be calculated directly using the built-in functions of “subBoundaryLength” and “area” in the MTEX toolbox version 5.5 and above. This fact suggests that SGB density could be used as a convenient metric to estimate stress in future and it might also avoid the problem caused by direction of profiles to measure intercept length (see below), although their underlying physics is essentially the same.

### Cautions of employing experimentally calibrated piezometers

Besides the effect of the step size as mentioned above, many other parameters can also play important roles in deriving stress using subgrain-related or EBSD-based piezometers. First, the estimation of intercept length and SGB density is significantly affected by the choice of misorientation threshold that defines LAMB or SGB. A lower threshold results in a higher LAMB or SGB density and vice versa (Supplementary Fig. S4). Second, the choices of denoising filters and boundary smoothing also affect the appearance of LAMBs or SGBs^55,56^. Third, the direction of profiles (e.g., parallel to either X- or Y-axes or random in the specimen reference frame^19,57,58^) can also yield contrasting intercept lengths of neighboring LAMBs or SGBs, especially when LAMBs or SGBs tend to have a preferred direction like our sample (Fig. 1e and Supplementary Fig. S2d). In addition, more factors can affect the measurement of microstructures and the estimation of stress by employing other piezometers, including segmentation of recrystallized grains, calculation of dislocation density, and selection of analytical parameters (e.g., kernal size of KAM), as well as the effects of structural factors (e.g., grain pinning, grain-to-grain interaction, distribution heterogeneity in substructures, and sampling size)^34,37,59,60^. Because of the appreciable influences of various factors listed above, to derive a reliable stress for natural samples, it is optimal to apply the same parameters and similar structures that are originally used to calibrate piezometers.

Despite the differences of parameters in calculating stress between those used in our study and those by Goddard, et al.^19^, their underlying principles are basically the same and our results still support the close relations between LAMB or SGB density, KAM, and stress. These correlations are built based on one natural sample and available subgrain-size piezometers, which may incur uncertainties and inaccuracies in the stress estimation. To derive more reliable piezometers based on SGB density and KAM, further calibrations using the experimentally deformed samples are needed.

## Methods

### Sample preparation

Owing to the prevalence of large grains, the sample 13LQ117 was cut into a large (12 × 6 cm^2^), thick (~ 150 μm) thin section in the XZ-plane, i.e., normal to the foliation (Z-direction) and parallel to the lineation (X-direction). The foliation and lineation were determined by the alignments of trails and shape preferred orientation of spinel in three nearly perpendicular sections. To obtain high quality electron backscattered patterns (EBSPs), the thin section was finally polished with an aqueous suspension containing 0.05 μm colloidal silica for > 2 h using a Buehler vibration polisher.

### EBSD data acquisition and treatment

Mineral phase and crystallographic orientation data are measured using an Oxford Symmetry EBSD detector attached to a Zeiss Sigma 300 field emission scanning electron microscope (FE-SEM) housed at the China University of Geosciences (Wuhan), China. An accelerating voltage of 15 kV, a spot size of 6, a beam current of 6 nA, and working distance of 16–23 mm were used. We mapped a large area (~ 4.3 × 1.8 cm^2^) with EBSD with a step size of 15 μm and small areas for 22 single olivine grains (without obvious dynamic recrystallization) with a step size of 1 μm (Supplementary Fig. S5 and Supplementary raw EBSD data). To examine the effect of step size on the derived microstructures at the same domains, the step size was then undersampled from 1 to 30 μm by selecting the points at regular row and column intervals in the 1 μm step-sized gridified EBSD data. The microstructures produced by artificially enlarged step sizes are very similar to those measured using various actual step sizes. The raw indexation rates for all mapping are larger than 95%. The raw EBSD data are cleaned by removing wild spikes (isolated pixels) and points with MAD > 0.8°. The denoising process is performed by applying Kuwahara filter with number of neighbors of 5, because this filter does not smooth out low-angle boundaries^47,55^.

The strength of the crystallographic preferred orientation (CPO) is quantified using M- and J-indices^61,62^. The M- and J-indices range from 0 to 1 and from 1 to infinity for random and single crystal fabrics, respectively. The BA-index is used to describe the olivine CPO symmetry^63^. This index classifies olivine CPOs into three types: (1) fiber-[010] ([010]-axis point plus [100]- and [001]-axes girdles, BA-index < 0.33), (2) orthorhombic ([100]-, [010]- and [001]-axes points, 0.33 < BA-index < 0.66), and (3) fiber-[100] ([100]-axis point plus [010] and [001]-axes girdles, BA-index > 0.66). The CPOs of olivine (Ol), orthopyroxene (Opx) and clinopyroxene (Cpx) are presented using one point per grain and shown in the pole figures.

A grain boundary is a high-angle boundary having misorientation angles larger than 10°–15° when defined using EBSD data^64,65^. To minimize the mistake of taking grain boundaries in the misorientation angle range of 10°–15° as low-angle boundaries, we selected the lower bound definition for a high-angle boundary, namely 10°, as the threshold to construct grain boundaries. In this case, a low-angle boundary can be defined as a boundary whose misorientation angle is greater than 1° and less than 10°. The choice of a lower limits of 1° (larger than the angular resolution of ~ 0.5° for our Symmetry EBSD system) for low-angle boundary and 10° for high-angle boundary is also adopted by many previous olivine and pyroxene studies e.g.^19,45,57,66^. To avoid the staircase effect generated during the grain reconstruction routine, grain boundaries and LAMBs are smoothed iteratively by 8 times.

Grains consisting of less than 10 pixels are omitted in the grain construction only for large-area EBSD map in which undersampling is not applied. The apparent grain size is reported as equivalent circular diameter and area-weighted mean grain size is calculated. The sinuosity of grain boundaries is quantified by the shape factor—the ratio between the actual perimeter of the grain and the perimeter of a circle with the same area. The ellipticity of grain is measured by aspect ratios—the length ratio of long to short axes of a fitted ellipse. The discrimination between twist and tilt LAMBs and inference of dislocation slip systems in olivine are based on the geometrical relations between misorientation axes, direction of LAMB trace, and olivine axis orientations at the LAMBs^67^. To quantify the degree of intracrystalline lattice distortion, M2M, GOS and KAM are also calculated.

Linear intercept length represents the distance between two neighboring LAMBs. Because of the low precision and large EBSD data volume, we did not calculate the linear-intercept length for the large-area mapping data. Instead, linear-intercept lengths are only calculated for the high spatial resolution ESBD maps of 22 olivine grains. For the olivine grains that show an obvious oblique alignment of LAMBs with respect to the horizontal direction, we rotated their EBSD data to make most of their LAMBs align vertically. Since most LAMBs are (sub)perpendicular to the horizontal X-axis after the rotation of EBSD data and they are parallel to each other, only X-axis parallel profiles are chosen. Along each profile, the points of intersection with the LAMB are detected and the intercept length between neighboring intersections is measured and recorded for further calculations (e.g., arithmetic mean of intercept length). Different from the method used by Goddard, et al.^19^, all X-axis parallel profile lines, which correspond to all rows in the gridified EBSD data, are used to calculate the intercept length. The complete number of intercept lines used in our study averts the unstable mean intercept length which varies significantly if the number of intercept lines is insufficient^19^. All above-mentioned EBSD data treatments were performed using the MTEX toolbox (ver. 5.5) in MATLAB (http://mtex-toolbox.github.io/)^68–70^.

### Stress estimation

In this study, three olivine subgrain-size piezometers are adopted to calculate stress, including an old version proposed by Toriumi^20^ and two new versions developed by Goddard, et al.^19^. The mean intercept length of LAMBs is used as a substitute for subgrain size in the formulae of piezometers. The new subgrain-size piezometers proposed by Goddard, et al.^19^ consider the cases with and without the correction of Holyoke and Kronenberg^71^. This correction accounts for the increased friction resulting from the Poisson effect on load column during specimen loading. For convenience, the three piezometers hereafter are abbreviated as T1979, G2020wHK2010, and G2020w/oHK2010.

It is noteworthy that differential stress is used by T1979 and von Mises equivalent stress is employed by G2020wHK2010 and G2020w/oHK2010. Because the geometry of the deformation experiment that T1979 adopted is axial compression^7^, differential stress is thus equal to von Mises equivalent stress^72^. Therefore, von Mises equivalent stress (in short, stress) is consistently applied in the three piezometers.

## Supplementary Information

Supplementary Information 1.

Supplementary Information 2.

Supplementary Figures.

Supplementary Information 4.

## Data Availability

All data generated or analyzed during this study are included in this published article and its supplementary files.

## References

[1] Twiss R (1977). Theory and applicability of a recrystallized grain size paleopiezometer. PAGEOPH.

[2] Heidbach O (2018). The World Stress Map database release 2016: Crustal stress pattern across scales. Tectonophysics.

[3] Bessat A, Duretz T, Hetényi G, Pilet S, Schmalholz SM (2020). Stress and deformation mechanisms at a subduction zone: Insights from 2-D thermomechanical numerical modelling. Geophys. J. Int..

[4] Bürgmann R, Dresen G (2008). Rheology of the lower crust and upper mantle: Evidence from rock mechanics, geodesy, and field observations. Annu. Rev. Earth Pl Sci..

[5] Wang Y, Liebermann R, Boland J (1988). Olivine as an in situ piezometer in high pressure apparatus. Phys. Chem. Minerals.

[6] Twiss, R. J. in *Mineral and Rock Deformation: Laboratory Studies Geophysical Monograph Series* 247–261 (1986).

[7] Kohlstedt DL, Goetze C (1974). Low-stress high-temperature creep in olivine single crystals. J. Geophys. Res..

[8] Karato S, Jung H (2003). Effects of pressure on high-temperature dislocation creep in olivine. Philos. Mag..

[9] Van der Wal D, Chopra P, Drury M, Gerald JF (1993). Relationships between dynamically recrystallized grain size and deformation conditions in experimentally deformed olivine rocks. Geophys. Res. Lett..

[10] Ross JV, Ave Lallemant HG, Carter NL (1980). Stress dependence of recrystallized-grain and subgrain size in olivine. Tectonophysics.

[11] Karato S-I, Toriumi M, Fujii T (1980). Dynamic recrystallization of olivine single crystals during high-temperature creep. Geophys. Res. Lett..

[12] Platt JP, De Bresser JHP (2017). Stress dependence of microstructures in experimentally deformed calcite. J. Struct. Geol..

[13] Siemes H, Rybacki E, Klingenberg B, Rosière CA (2011). Development of a recrystallized grain size piezometer for hematite based on high-temperature torsion experiments. Eur. J. Mineral..

[14] Linckens J, Bruijn RHC, Skemer P (2014). Dynamic recrystallization and phase mixing in experimentally deformed peridotite. Earth Planet. Sci. Lett..

[15] Bruijn RHC, Skemer P (2014). Grain-size sensitive rheology of orthopyroxene. Geophys. Res. Lett..

[16] Cross AJ, Prior DJ, Stipp M, Kidder S (2017). The recrystallized grain size piezometer for quartz: An EBSD-based calibration. Geophys. Res. Lett..

[17] Stipp M, Tullis J, Scherwath M, Behrmann JH (2010). A new perspective on paleopiezometry: Dynamically recrystallized grain size distributions indicate mechanism changes. Geology.

[18] Stipp M (2003). The recrystallized grain size piezometer for quartz. Geophys. Res. Lett..

[19] Goddard RM (2020). A subgrain-size piezometer calibrated for EBSD. Geophys. Res. Lett..

[20] Toriumi M (1979). Relation between dislocation density and subgrain size of naturally deformed olivine in peridotites. Contrib. Miner. Petrol..

[21] Durham WB, Goetze C, Blake B (1977). Pastic-flow of oriented single-crystals of olivine. 2. Observations and interpretations of dislocation-structures. J. Geophys. Res..

[22] Mercier J-CC, Anderson DA, Carter NL (1977). Stress in the lithosphere: Inferences from steady state flow of rocks. Pure Appl. Geophys. PAGEOPH.

[23] Friedman, M. & Higgs, N. G. in *Mechanical Behavior of Crustal Rocks* 11–27 (1981).

[24] Karato S (1987). Scanning electron microscope observation of dislocations in olivine. Phys. Chem. Minerals.

[25] Hirsch PB, Howie A, Whelan MJ (1960). A kinematical theory of diffraction contrast of electron transmission microscope images of dislocations and other defects. Philos. Trans. R. Soc. Lond. Ser. A Math. Phys. Sci..

[26] Green HW, Radcliffe SV (1972). Dislocation mechanisms in olivine and flow in the upper mantle. Earth Planet. Sci. Lett..

[27] Wallis D, Hansen LN, Ben Britton T, Wilkinson AJ (2016). Geometrically necessary dislocation densities in olivine obtained using high-angular resolution electron backscatter diffraction. Ultramicroscopy.

[28] Cao Y (2015). Plastic deformation and seismic properties in fore-arc mantles: A petrofabric analysis of the Yushigou Harzburgites, North Qilian Suture Zone, NW China. J. Petrol..

[29] Cao Y, Jung H, Song S (2017). Olivine fabrics and tectonic evolution of fore-arc mantles: A natural perspective from the Songshugou dunite and harzburgite in the Qinling orogenic belt, central China. Geochem. Geophys. Geosyst..

[30] De Kloe, R. *Deformation Mechanisms and Melt Nano-structures in Experimentally Deformed Olivine-Orthopyroxene Rocks with Low Melt Fractions: An Electron Microscopy Study* Ph.D. thesis, Utrecht University (2001).

[31] Passchier, C. W. & Trouw, R. A. J. *Microtectonics*, 2nd edn, 366 (Springer, 2005).

[32] Li Z, Wen D, Wang Y, Liu X (2020). An investigation of dislocation in olivine phenocrysts from the Hawaiian basalts. J. Earth Sci..

[33] Poirier, J. P. *Creep of Crystals: High-Temperature Deformation Processes in Metals, Ceramics, and Minerals* (Cambridge University Press, 1985).

[34] Lopez-Sanchez MA, Tommasi A, Ismail WB, Barou F (2021). Dynamic recrystallization by subgrain rotation in olivine revealed by electron backscatter diffraction. Tectonophysics.

[35] Karato S, Jung H, Katayama I, Skemer P (2008). Geodynamic significance of seismic anisotropy of the upper mantle: New insights from laboratory studies. Annu. Rev. Earth Planet Sci..

[36] Tielke J, Mecklenburgh J, Mariani E, Wheeler J (2019). The influence of water on the strength of olivine dislocation slip systems. J. Geophys. Res. Sol Earth.

[37] Ben Ismail W (2021). Deformation of upper mantle rocks with contrasting initial fabrics in axial extension. Tectonophysics.

[38] Tommasi A, Godard M, Coromina G, Dautria JM, Barsczus H (2004). Seismic anisotropy and compositionally induced velocity anomalies in the lithosphere above mantle plumes: A petrological and microstructural study of mantle xenoliths from French Polynesia. Earth Planet Sci. Lett..

[39] Le Roux V, Tommasi A, Vauchez A (2008). Feedback between melt percolation and deformation in an exhumed lithosphere–asthenosphere boundary. Earth Planet Sci. Lett..

[40] Soustelle V, Tommasi A, Demouchy S, Ionov DA (2010). Deformation and fluid–rock interaction in the supra-subduction mantle: Microstructures and water contents in peridotite xenoliths from the Avacha volcano, Kamchatka. J. Petrol..

[41] Mackwell SJ (1991). High-temperature rheology of enstatite—Implications for creep in the mantle. Geophys. Res. Lett..

[42] Raterron P, Fraysse G, Girard J, Holyoke CW (2016). Strength of orthoenstatite single crystals at mantle pressure and temperature and comparison with olivine. Earth Planet Sci. Lett..

[43] Jung H, Park M, Jung S, Lee J (2010). Lattice preferred orientation, water content, and seismic anisotropy of orthopyroxene. J. Earth Sci..

[44] Tommasi A, Vauchez A, Ionov DA (2008). Deformation, static recrystallization, and reactive melt transport in shallow subcontinental mantle xenoliths (Tok Cenozoic volcanic field, SE Siberia). Earth Planet Sci. Lett..

[45] Henry H (2017). Deformation of mantle pyroxenites provides clues to geodynamic processes in subduction zones: Case study of the Cabo Ortegal Complex, Spain. Earth Planet Sci. Lett..

[46] Manthilake MAGM, Miyajima N, Heidelbach F, Soustelle V, Frost DJ (2013). The effect of aluminum and water on the development of deformation fabrics of orthopyroxene. Contrib. Mineral. Petrol..

[47] Humphreys FJ (2001). Review—Grain and subgrain characterisation by electron backscatter diffraction. J. Mater. Sci..

[48] Wright SI, Nowell MM, Field DP (2011). A review of strain analysis using electron backscatter diffraction. Microsc. Microanal..

[49] Hidas K (2019). Lithosphere tearing along STEP faults and synkinematic formation of lherzolite and wehrlite in the shallow subcontinental mantle. Solid Earth Discuss..

[50] Rui S-S, Niu L-S, Shi H-J, Wei S, Tasan CC (2019). Diffraction-based misorientation mapping: A continuum mechanics description. J. Mech. Phys. Solids.

[51] Calcagnotto M, Ponge D, Demir E, Raabe D (2010). Orientation gradients and geometrically necessary dislocations in ultrafine grained dual-phase steels studied by 2D and 3D EBSD. Mater. Sci. Eng. A.

[52] Wallis D, Hansen LN, Britton TB, Wilkinson AJ (2019). High-angular resolution electron backscatter diffraction as a new tool for mapping lattice distortion in geological minerals. J. Geophys. Res. Solid Earth.

[53] Samae V (2021). Stress-induced amorphization triggers deformation in the lithospheric mantle. Nature.

[54] Wallis D (2020). Dislocation interactions during low-temperature plasticity of olivine and their impact on the evolution of lithospheric strength. Earth Planet. Sci. Lett..

[55] Hielscher R, Silbermann CB, Schmidl E, Ihlemann J (2019). Denoising of crystal orientation maps. J. Appl. Crystallogr..

[56] Fan S (2021). Using grain boundary irregularity to quantify dynamic recrystallization in ice. Acta Mater..

[57] Hansen LN, Warren JM (2015). Quantifying the effect of pyroxene on deformation of peridotite in a natural shear zone. J. Geophys. Res. Solid Earth.

[58] Valcke SLA, Pennock GM, Drury MR, De Bresser JHP (2006). Electron backscattered diffraction as a tool to quantify subgrains in deformed calcite. J. Microsc..

[59] Lopez-Sanchez MA (2020). Which average, how many grains, and how to estimate robust confidence intervals in unimodal grain size populations. J. Struct. Geol..

[60] Huang K, Logé RE (2016). A review of dynamic recrystallization phenomena in metallic materials. Mater. Des..

[61] Skemer P, Katayama B, Jiang ZT, Karato S (2005). The misorientation index: Development of a new method for calculating the strength of lattice-preferred orientation. Tectonophysics.

[62] Bunge, H. *Texture Analysis in Materials Science: Mathematical Models*, 593 (Butterworths, 1982).

[63] Mainprice D, Bachmann F, Hielscher R, Schaeben H (2015). Descriptive tools for the analysis of texture projects with large datasets using MTEX: Strength, symmetry and components. Geol. Soc. Lond. Spec. Publ..

[64] Trimby PW, Prior DJ, Wheeler J (1998). Grain boundary hierarchy development in a quartz mylonite. J. Struct. Geol..

[65] Karato, S. *Deforamtion of Earth Materials—An introduction to the Rheology of Solid Earth* (Cambridge University Press, 2008).

[66] Ambrose TK, Wallis D, Hansen LN, Waters DJ, Searle MP (2018). Controls on the rheological properties of peridotite at a palaeosubduction interface: A transect across the base of the Oman–UAE ophiolite. Earth Planet Sci. Lett..

[67] Wieser PE, Edmonds M, Maclennan J, Wheeler J (2020). Microstructural constraints on magmatic mushes under Kilauea Volcano, Hawai'i. Nat. Commun..

[68] Bachmann F, Hielscher R, Schaeben H (2010). Texture analysis with MTEX—Free and open source software toolbox. Solid State Phenom..

[69] Bachmann F, Hielscher R, Schaeben H (2011). Grain detection from 2d and 3d EBSD data—Specification of the MTEX algorithm. Ultramicroscopy.

[70] Hielscher R, Schaeben H (2008). A novel pole figure inversion method: Specification of the MTEX algorithm. J. Appl. Crystallogr..

[71] Holyoke CW, Kronenberg AK (2010). Accurate differential stress measurement using the molten salt cell and solid salt assemblies in the Griggs apparatus with applications to strength, piezometers and rheology. Tectonophysics.

[72] Paterson MS, Olgaard DL (2000). Rock deformation tests to large shear strains in torsion. J. Struct. Geol..

