# Supplementary material for: Intracrystalline deformation microstructures in natural olivine with implications for stress estimation
Source: Sci Rep. 2022 Nov 22;12:20069. doi: 10.1038/s41598-022-24538-2 (PMC9681765; doi:10.1038/s41598-022-24538-2)
Supplement: Supplementary file 1 — Supplementary Information 1. [file 41598_2022_24538_MOESM1_ESM.pdf]

Intracrystalline deformation microstructures in natural olivine with implications for stress estimation

Jian Ma[1], Wenlong Liu[1], Yi Cao[1,*], Junfeng Zhang[1], and Chuanzhou Liu[2,3,4]

[1] State Key Laboratory of Geological Processes and Mineral Resources, School of Earth Sciences, China University of Geosciences, Wuhan 430074, China
[2] State Key Laboratory of Lithospheric Evolution, Institute of Geology and Geophysics, Chinese Academy of Sciences, Beijing 100029, China
[3] CAS Center for Excellence in Tibetan Plateau Earth Sciences, Beijing 100101, China
[4] University of Chinese Academy of Sciences, Beijing 100049, China

*Correspondence to:
Yi Cao (caoyi0701@126.com)

Supplementary text

Geological background and sample description

The Yarlung Tsangbo suture zone (YTSZ), separating the Eurasian plate to the north and the Indian plate to the south, can be geographically divided into three segments from west to east—Kiogar-Saga, Sangsang-Dazhuqu, and Zedang [1]. A series of ophiolites are exposed discontinuously along the YTSZ (Fig. A1a), which have been commonly regarded as relics of the Neo-Tethys Ocean [2]. Hitherto, tectonic settings proposed for the YTS ophiolites can be classified into a subduction-related group, including backarc, forearc, backarc to forearc and volcanic arc settings, and a subduction-unrelated group, including mid-ocean ridge, continental margin, and mantle plume settings [3].

The Xigaze ophiolites are located ~300 km west of Lhasa, belonging to the central segment of YTSZ (Fig. A1a) [4,5]. The Xigaze ophiolites are composed of several renowned sections, including Dazhuqu, Bainang, Luqu (also called as the Beimarang, this study), Qunrang, Jiding and Lhaze, which are named after the nearby villages. The Xigaze ophiolite sequence is characterized by a thin crustal sequence (with a thickness of less than 2 km) but a thick mantle section that consists mainly of spinel-bearing harzburgite [6-8]. The Luqu ophiolite is part of the Xigaze ophiolites [9]. Resembling the other Xigaze ophiolites, Luqu ophiolites also have a thick mantle section in the south and a thin oceanic crust section in the north (Fig. A1b). The southernmost part exposes a nearly continuous ophiolitic mélange with an average thickness of ~1 km, where several mafic rock blocks are observed, some of which display strongly foliated textures [10,11]. To the north, serpentinite with a few mafic blocks gradually becomes the main body. The fresh peridotite crops out locally on the top of the serpentinite. The peridotite

mainly consists of harzburgite with a minor amount of dunite and lherzolite, together with fresh pyroxenites and coarse-grained gabbroic dyke intrusions [4,10].

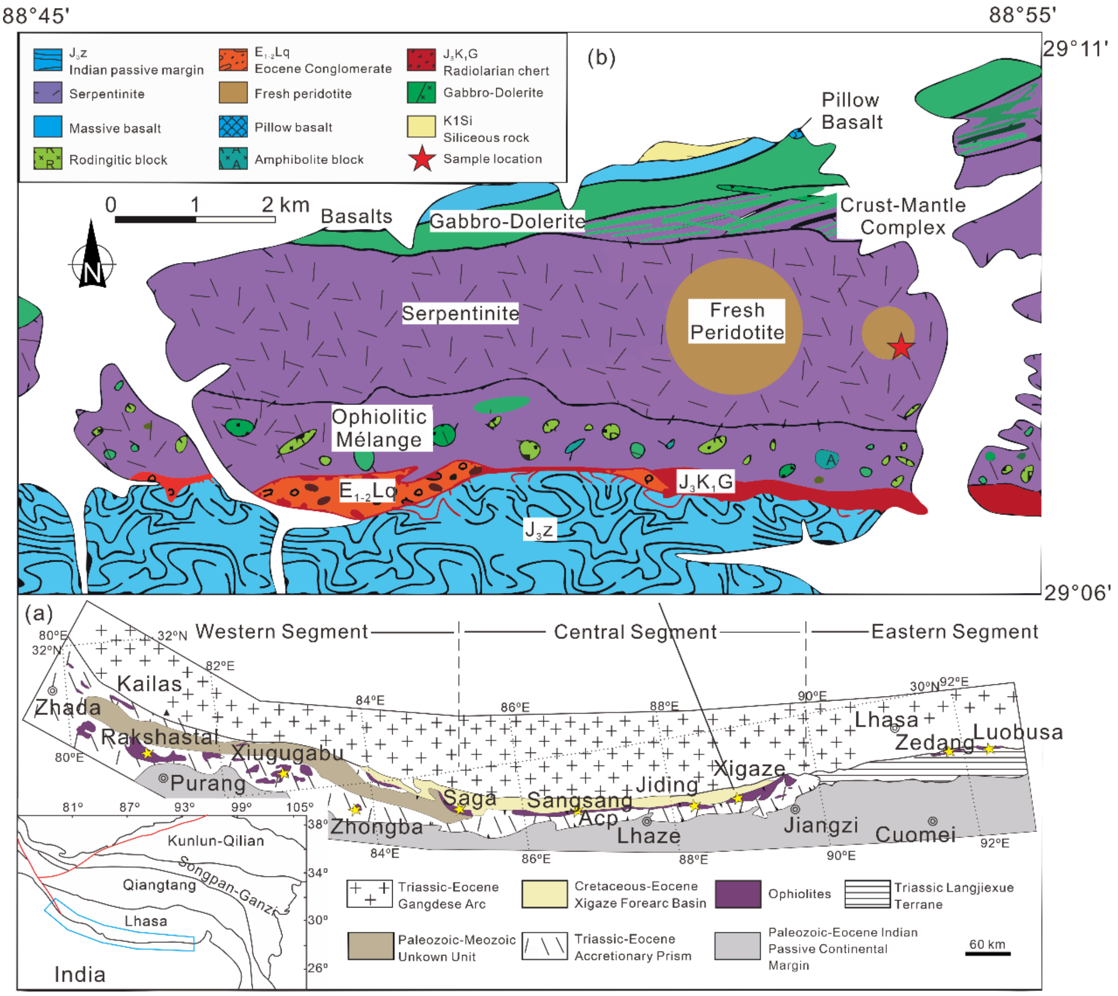

Figure A1. Simplified geological maps showing the (a) Yarlung Tsangbo suture zone (YTSZ) and the (b) Luqu ophiolite sequence (modified after Zhang, et al. [4]). Red stars indicate the sample location.

One fresh harzburgite, sample 13LQ117, was selected for this study (Figs. A1b and 1). Like other Luqu peridotites, pyroxenes occur as veins, crosscutting the peridotite foliation obliquely. A previous study by Zhang, et al. [4] suggests that this harzburgite sample is characterized by an (ultra-)refractory composition, with respective SiO_2 and MgO contents of 41.03 and 49.49 wt.%, whole-rock Mg# and Cr# of 0.91 and 0.51, a high olivine Mg# of 0.9, and a high spinel Cr# of 0.62. Besides, clinopyroxene exhibits variable depletion in the light rare earth elements.

GOS is NOT a viable piezometer

Grain orientation spread (GOS) or average mis-to mean (M2M), i.e., the mean misorientation angle between every pixel and their mean orientation in a grain, reflects the average degree of lattice distortion of the grain. GOS can increase owing to the accumulation of geometrically necessary dislocations (GNDs) and decrease due to annihilation of GNDs by static recrystallization (i.e., annealing or recovery) e.g.[12,13-16]. Because GOS is expected to be controlled by the density of GND, it may correlate positively with stress accordingly. If this correlation holds, it suggests that it is possible to use GOS as a piezometer to estimate stress, which is more convenient.

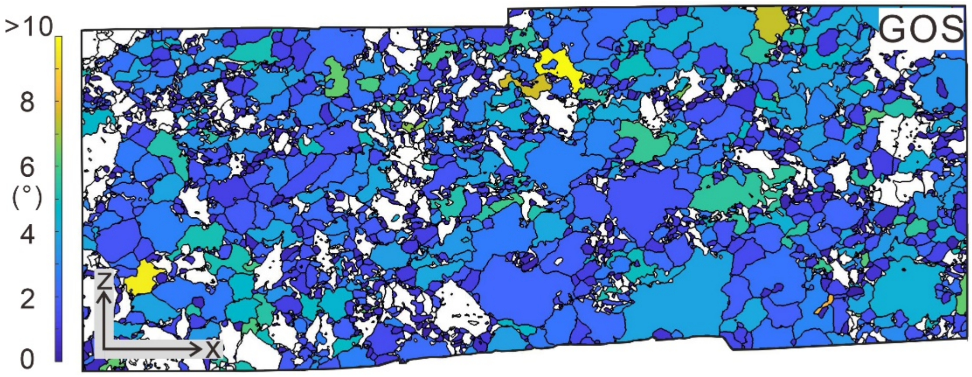

Figure A2. GOS map of olivine grains in sample 13LQ117. EBSD data were collected using the step size of 15 μm. GOS is calculated using MTEX toolbox (ver. 5.5) in MATLAB (http://mtex-toolbox.github.io/) [17-19].

Similar to M2M and KAM, GOS is unevenly distributed in olivines (Fig. A2) and overall much higher than that in orthopyroxenes (Supplementary Fig. S3b). As the step size changes, GOS varies in the largely overlapped ranges of 1.17−3.14°, 1.90−4.61° and 1.82−4.51° in low-, medium- and high-ρ_{LAMB} grains, respectively, suggesting the absence of correlation between GOS and LAMB density (Fig. A3a and Supplementary Table S2). Besides, GOS is almost invariable at the step sizes less than 15 μm, while more significant fluctuations are observed at the step size larger than 15 μm (Fig. A3a). For the large-area mapping data, average GOS ranges from 0.1° to 2.7°, with an area-weighted mean of 2.81° (grey area and green star in Fig. A3a). This range of GOS is only partly overlapped with that of the 22 olivine grains produced at the step size of 15

μm.

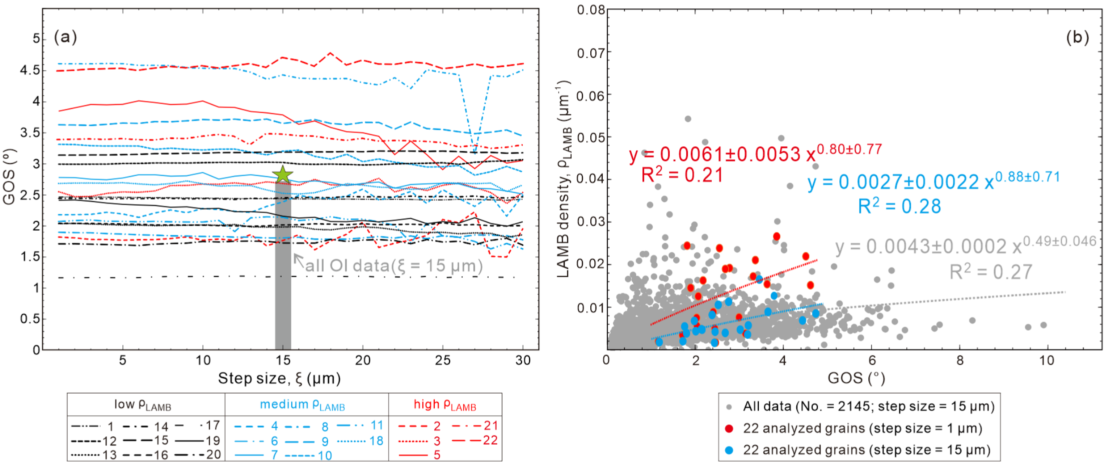

Figure A3. (a) Variations of GOS with step sizes from 1 to 30 μm in the 22 analyzed olivine grains, which are classified into low-, medium- and high-ρ_{lamb} grains. For the large-area EBSD mapping data using the step size of 15 μm, the grey bar is the range of arithmetic mean plus and minus standard deviation, and the green stars are area-weighted averages. (b) LAMB density vs. GOS. The colors of the fitting curves and the fitting equation correspond to the colors of scatters.

Besides, GOS fluctuates much less remarkably with step size, especially at the step size ranging from 1 to 15 μm (Fig. A3a). This result implies that we can obtain a relatively stable GOS by using a relatively large step size (e.g., ≥15 μm), especially for EBSD mapping of natural samples, due to the large analyzed area. However, for step sizes of 1 and 15 μm, the correlations between GOS and LAMB density (R^2 = 0.21 and 0.28, Fig. A3b) are very weak. The correlations between GOS and stress are also weak (R^2 = 0.2 and 0.41, Figs. A4a and b), although only the stress values derived from the minimum step size of 1 μm, where LAMBs are predominantly SGBs, are most likely valid.

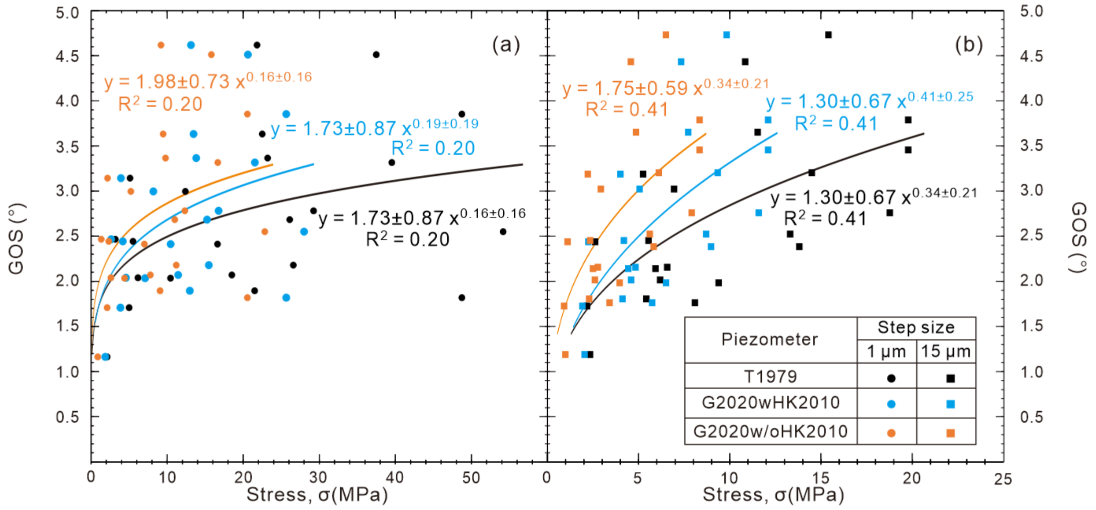

Figure A4. Relations between GOS and stress for the 22 analyzed olivine grains at the step size of 1 μm (a, circles, left column) and 15 μm (b, rectangles, right column).

These results indicate that the degree of LAMB development may have no significant relation with GOS. In other words, for grains with high GOS, the density of LAMB can be smaller, and vice versa. This fact can also be observed in the distributions of GOS with step size, in which GOS is not correlated with the grains of different LAMB density (Fig. A3a). The reason for this anti-correlation is that the GOS of a grain is not only related to the misorientation angle between adjacent pixels, but is also affected by the orientation distribution of all pixels in the grain. An explanatory example is given in Fig. A5, in which the cases of high LAMB density and small GOS (Fig. A5a) and of low LAMB density and large GOS (Fig. A5b) are shown. The weak correlations between GOS and LAMB density, as well as between GOS and stress suggest that GOS is not a reliable metric to estimate stress.

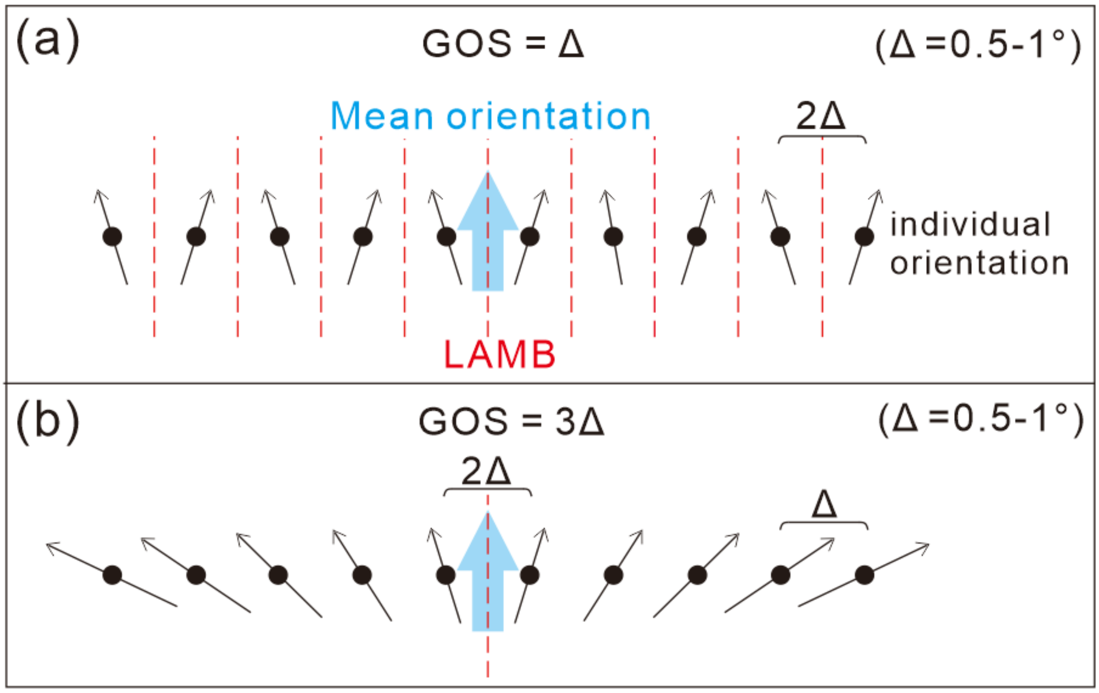

Figure A5. Schematic diagrams showing the orientations of 10 individual points (black arrows), and the resultant LAMBs (red dashed lines) and GOS in two grains. The mean orientations of two grains are indicated by cyan arrows, which are the same in the two grains, because of their symmetric distributions of individual points across the profiles. In (a), greater misorientation between neighboring points ($2\Delta = 1{-}2°$) results in 9 parallelly aligned LAMBs, whereas GOS is small ($1\Delta = 0.5{-}1°$). In (b), smaller misorientation ($1\Delta = 0.5{-}1°$) between individual points in both left and right halves of the grains produces null LAMB, and only one LAMB occurs in the center of the grain, due to the larger misorientation ($2\Delta = 1{-}2°$) between the two neighboring grains here. The gradually increasing misorientations of individual points relative to their mean orientation away from the center form a high GOS of 3Δ (1.5−3°).

References

1 Hébert, R. *et al.* The Indus–Yarlung Zangbo ophiolites from Nanga Parbat to Namche Barwa syntaxes, southern Tibet: First synthesis of petrology, geochemistry, and geochronology with incidences on geodynamic reconstructions of Neo-Tethys. *Gondwana Research* **22**, 377-397, doi:10.1016/j.gr.2011.10.013 (2012).

2 Zhang, C., Liu, C.-Z., Liu, T. & Wu, F.-Y. Evolution of mantle peridotites from the Luobusa ophiolite in the Tibetan Plateau: Sr-Nd-Hf-Os isotope constraints. *Lithos* **362-363**, doi:10.1016/j.lithos.2020.105477 (2020).

3 Furnes, H., Dilek, Y., Zhao, G., Safonova, I. & Santosh, M. Geochemical characterization of ophiolites in the Alpine-Himalayan Orogenic Belt: Magmatically and tectonically diverse evolution of the Mesozoic Neotethyan oceanic crust. *Earth-Science Reviews* **208**, doi:10.1016/j.earscirev.2020.103258 (2020).

4 Zhang, C. *et al.* Ultra-refractory mantle domains in the Luqu ophiolite (Tibet): Petrology and

tectonic setting. *Lithos* **286-287**, 252-263, doi:10.1016/j.lithos.2017.05.021 (2017).

5 Wu, F.-Y., Ji, W.-Q., Liu, C.-Z. & Chung, S.-L. Detrital zircon U–Pb and Hf isotopic data from the Xigaze fore-arc basin: Constraints on Transhimalayan magmatic evolution in southern Tibet. *Chemical Geology* **271**, 13-25, doi:10.1016/j.chemgeo.2009.12.007 (2010).

6 Girardeau, J., Mercier, J. C. C. & Zao, Y. O. Structure of the Xigaze Ophiolite, Yarlung Zangbo Suture Zone, Southern Tibet, China - Genetic-Implications. *Tectonics* **4**, 267-&, doi:10.1029/TC004i003p00267 (1985).

7 Girardeau, J., Mercier, J. C. C. & Yougong, Z. Origin of the Xigaze Ophiolite, Yarlung Zangbo Suture Zone, Southern Tibet. *Tectonophysics* **119**, 407-433, doi:10.1016/0040-1951(85)90048-4 (1985).

8 Nicolas, A., Girardeau, J., Marcoux, J., Dupre, B., Wang, X.B., Cao, Y.G., . The Xigaze ophiolite (Tibet): a peculiar oceanic lithosphere. *nature* (1981).

9 Zhao, M.-S., Chen, Y.-X. & Zheng, Y.-F. Geochemical evidence for forearc metasomatism of peridotite in the Xigaze ophiolite during subduction initiation in Neo-Tethyan Ocean, south to Tibet. *Lithos* **380-381**, doi:10.1016/j.lithos.2020.105896 (2021).

10 Zhang, C., Liu, C.-Z., Ji, W.-B., Liu, T. & Wu, F.-Y. Heterogeneous sub-ridge mantle of the Neo-Tethys: Constraints from Re-Os isotope and HSE compositions of the Xigaze ophiolites. *Lithos* **378-379**, doi:10.1016/j.lithos.2020.105819 (2020).

11 Huot, F. o. The Beimarang me´lange (southern Tibet) brings additional constraints in assessing the origin, metamorphic evolution and obduction processes of the Yarlung Zangbo ophiolite. (2002).

12 Hidas, K. *et al.* Flow in the western Mediterranean shallow mantle: Insights from xenoliths in Pliocene alkali basalts from SE Iberia (eastern Betics, Spain). *Tectonics* **35**, 2657-2676, doi:10.1002/2016tc004165 (2016).

13 Cross, A. J., Prior, D. J., Stipp, M. & Kidder, S. The recrystallized grain size piezometer for quartz: An EBSD-based calibration. *Geophys Res Lett* **44**, 6667-6674, doi:10.1002/2017gl073836 (2017).

14 Demouchy, S., Tommasi, A., Ionov, D., Higgie, K. & Carlson, R. W. Microstructures, water contents, and seismic properties of the mantle lithosphere beneath the northern limit of the Hangay Dome, Mongolia. *Geochem Geophys Geosyst* **20**, 183-207, doi:10.1029/2018gc007931 (2019).

15 Nzogang, B. C., Thieme, M., Mussi, A., Demouchy, S. & Cordier, P. Characterization of recovery onset by subgrain and grain boundary migration in experimentally deformed polycrystalline olivine. *Eur J Mineral* **32**, 13-26, doi:10.5194/ejm-32-13-2020 (2020).

16 Tommasi, A., Baptiste, V., Vauchez, A. & Holtzman, B. Deformation, annealing, reactive melt percolation, and seismic anisotropy in the lithospheric mantle beneath the southeastern Ethiopian rift: Constraints from mantle xenoliths from Mega. *Tectonophysics* **682**, 186-205, doi:10.1016/j.tecto.2016.05.027 (2016).

17 Bachmann, F., Hielscher, R. & Schaeben, H. Texture Analysis with MTEX – Free and Open Source Software Toolbox. *Solid State Phenom* **160**, 63-68, doi:10.4028/www.scientific.net/SSP.160.63 (2010).

18 Bachmann, F., Hielscher, R. & Schaeben, H. Grain detection from 2d and 3d EBSD data—Specification of the MTEX algorithm. *Ultramicroscopy* **111**, 1720-1733, doi:10.1016/j.ultramic.2011.08.002 (2011).

19 Hielscher, R. & Schaeben, H. A novel pole figure inversion method: specification of the MTEX algorithm. *J Appl Crystallogr* **41**, 1024-1037, doi:10.1107/S0021889808030112 (2008).
