# Supplementary material for: Intracrystalline deformation microstructures in natural olivine with implications for stress estimation
Source: Sci Rep. 2022 Nov 22;12:20069. doi: 10.1038/s41598-022-24538-2 (PMC9681765; doi:10.1038/s41598-022-24538-2)
Supplement: Supplementary file 2 — Supplementary Information 2. [file 41598_2022_24538_MOESM2_ESM.pdf]

Intracrystalline deformation microstructures in natural olivine with implications for stress estimation

Jian Ma[1], Wenlong Liu[1], Yi Cao[1,*], Junfeng Zhang[1], and Chuanzhou Liu[2,3,4]

[1] State Key Laboratory of Geological Processes and Mineral Resources, School of Earth Sciences, China University of Geosciences, Wuhan 430074, China
[2] State Key Laboratory of Lithospheric Evolution, Institute of Geology and Geophysics, Chinese Academy of Sciences, Beijing 100029, China
[3] CAS Center for Excellence in Tibetan Plateau Earth Sciences, Beijing 100101, China
[4] University of Chinese Academy of Sciences, Beijing 100049, China

*Correspondence to:
Yi Cao (caoyi0701@126.com)

Table S1. Mineral area proportion, microstructural, and crystallographic preferred orientation parameters in sample 13LQ117.

Phase	Area proportion (%)	J-index	M-index	Grain size[1] (μm)	Shape factor[2]	Aspect ratio[2]	GOS[2] (°)	LAMB density[2] (μm^{-1})
Olivine	78.5	2.46	0.12	1496	1.57	1.65	2.81	0.0044
Orthopyroxene	20.0	1.43	0.02	1007	1.65	1.72	1.51	0.0017
Clinopyroxene	1.3	2.53	0.03	170	1.37	1.85	0.64	0.0022
Spinel[3]	0.2	-	-	311	1.50	1.94	1.23	0.0037

[1] Average 2D grain size weighted by the grain area.
[2] Average parameters weighted by grain area.
[3] J- and M-indices of spinel were not calculated due to limited number of grains.

Table S2. Summarized results in 22 selected olivine grains.

Grain classifica- -tions [1]	No.	GOS[2] (°)	Median KAM[2] (°)	LAMB density[2] (μm⁻¹)	Mean intercept length[2] (μm)	Grain size[2,3] (μm)	Mean differential stress (MPa): T1997[4] 1μm	T1997[4] 15μm	G2020wHK2010[5] 1μm	G2020wHK2010[5] 15μm	G2020w/oHK2010[6] 1μm	G2020w/oHK2010[6] 15μm	Dislocation slip system proportion (%): (010) [100]	(010) [001]	(100) [001]	{0kl} [100]	(001) [100]	LAMB type proportion (%): Tilt	Twist
Low ρ_{LAMB}	1	2.47	0.008	1.69E-03	458 (±414)	1744	3.2	2.6	3.1	2.6	1.6	1.3	13.2	1.3	1.3	34.8	49.5	68.7	31.3
	12	3.00	0.012	7.67E-03	118 (±176)	1087	12.4	6.9	9.5	5.8	6.0	3.4	3.5	0.8	2.5	52.3	41.0	99.9	0.1
	13	2.04	0.012	7.54E-03	140 (±131)	679	10.4	9.4	8.2	7.5	5.1	4.6	8.6	0.0	0.0	69.7	21.6	70.7	29.3
	14	2.44	0.011	5.09E-03	265 (±285)	938	5.5	5.5	4.8	4.8	2.7	2.7	55.7	5.6	1.5	22.3	14.9	98.9	1.1
	15	3.14	0.010	3.80E-03	285 (±237)	1023	5.1	5.2	4.5	4.6	2.5	2.6	28.6	0.4	1.7	28.6	40.7	92.9	7.1
	16	2.04	0.012	5.65E-03	237 (±226)	779	6.2	6.2	5.3	5.3	3.0	3.0	2.9	7.2	2.2	76.0	11.7	60.3	39.7
	17	1.17	0.007	1.96E-03	692 (±799)	723	2.1	2.3	2.2	2.3	1.0	1.1	0.3	1.2	0.3	42.8	55.4	99.0	1.0
	19	2.41	0.011	8.91E-03	88 (±101)	517	16.6	6.6	12.1	5.6	8.1	3.2	0.0	0.2	1.0	82.2	16.7	90.9	9.1
	20	1.71	0.010	3.48E-03	292 (±322)	927	5.0	2.2	4.4	2.2	2.4	1.1	30.2	0.1	9.1	51.2	9.4	91.1	8.9
Medium ρ_{LAMB}	4	2.18	0.015	1.64E-02	55 (±58)	278	26.6	13.8	17.9	10.3	12.9	6.7	0.0	0.0	1.2	59.9	38.8	49.8	50.2
	6	1.90	0.009	1.46E-02	68 (±104)	677	21.5	5.4	15.0	4.7	10.5	2.6	9.0	0.0	5.1	67.4	18.5	95.9	4.1
	7	2.78	0.019	1.93E-02	50 (±47)	228	29.3	18.8	19.4	13.4	14.2	9.1	10.4	1.1	0.4	22.6	65.6	90.0	10.0
	8	4.61	0.019	1.53E-02	67 (±66)	513	21.8	10.8	15.2	8.5	10.6	5.3	19.0	4.0	0.4	59.4	17.1	85.7	14.3
	9	3.6	0.021	1.55E-02	65 (±69)	648	22.5	11.5	15.6	8.9	11.0	5.6	32.8	2.4	1.2	40.1	23.6	84.0	16.0
	10	3.32	0.029	1.74E-02	37 (±)21	618	39.5	14.5	24.9	10.8	19.2	7.0	9.2	1.0	0.3	69.0	20.4	80.8	19.2
	11	2.07	0.009	1.27E-02	79 (±63)	392	18.5	5.9	13.2	5.1	9.0	2.9	0.0	0.1	0.0	98.3	1.7	66.6	33.4
	18	2.68	0.020	1.91E-02	56 (±57)	260	26.1	13.3	17.6	10.0	12.7	6.5	5.0	0.0	0.0	71.4	23.6	64.6	35.4
High ρ_{LAMB}	2	1.82	0.016	2.47E-02	30 (±21)	190	48.8	8.1	29.6	6.6	23.7	3.9	8.0	0.0	0.2	90.9	0.9	88.6	11.4
	3	2.55	0.028	2.41E-02	27 (±16)	195	54.2	-	32.3	-	26.4	-	0.9	0.0	0.0	97.2	1.9	43.9	56.1
	5	3.9	0.018	2.66E-02	30 (±22)	358	48.8	19.8	29.6	14.0	23.7	9.6	0.0	0.0	3.3	5.1	91.6	41.1	58.9
	21	3.36	0.014	2.12E-02	63 (±63)	408	23.2	19.8	16.0	14.0	11.3	9.6	9.6	6.7	10.2	47.2	26.3	80.6	19.4
	22	4.51	0.016	2.21E-02	39 (±39)	320	37.5	15.4	23.8	11.3	18.3	7.5	13.1	0.0	0.3	53.4	33.2	86.9	13.1

[1] Grain classifications were classified by the density of LAMB in different grains.

[2] GOS, KAM, LAMB density, intercept length and grain size were calculated at the step size of 1 μm.

[3] Grain size is represented the by the diameter of a circle with equivalent area to that of the analyzed domain.

[4] The estimated stress using the subgrain-size piezometer from Toriumi [1].

[5] The estimated stress using the subgrain-size piezometer from Goddard, et al. [2] with the calibration of Holyoke Iii and Kronenberg [3].

[6] The estimated stress using the subgrain-size piezometer from Goddard, et al. [2] without the calibration of Holyoke Iii and Kronenberg [3].

References

1 Toriumi, M. Relation between dislocation density and subgrain size of naturally deformed olivine in peridotites. *Contributions to Mineralogy and Petrology* **68**, 181-186, doi:10.1007/bf00371899 (1979).

2 Goddard, R. M. *et al.* A Subgrain-Size Piezometer Calibrated for EBSD. *Geophysical Research Letters* **47**, doi:10.1029/2020gl090056 (2020).

3 Holyoke Iii, C. W. & Kronenberg, A. K. Accurate differential stress measurement using the molten salt cell and solid salt assemblies in the Griggs apparatus with applications to strength, piezometers and rheology. *Tectonophysics* **494**, 17-31, doi:10.1016/j.tecto.2010.08.001 (2010).
