# Supplementary material for: Intracrystalline deformation microstructures in natural olivine with implications for stress estimation
Source: Sci Rep. 2022 Nov 22;12:20069. doi: 10.1038/s41598-022-24538-2 (PMC9681765; doi:10.1038/s41598-022-24538-2)
Supplement: Supplementary file 3 — Supplementary Figures. [file 41598_2022_24538_MOESM3_ESM.pdf]

Intracrystalline deformation microstructures in natural olivine with implications for stress estimation

Jian Ma[1], Wenlong Liu[1], Yi Cao[1,*], Junfeng Zhang[1], and Chuanzhou Liu[2,3,4]

[1] State Key Laboratory of Geological Processes and Mineral Resources, School of Earth Sciences, China University of Geosciences, Wuhan 430074, China
[2] State Key Laboratory of Lithospheric Evolution, Institute of Geology and Geophysics, Chinese Academy of Sciences, Beijing 100029, China
[3] CAS Center for Excellence in Tibetan Plateau Earth Sciences, Beijing 100101, China
[4] University of Chinese Academy of Sciences, Beijing 100049, China

*Correspondence to:
Yi Cao (caoyi0701@126.com)

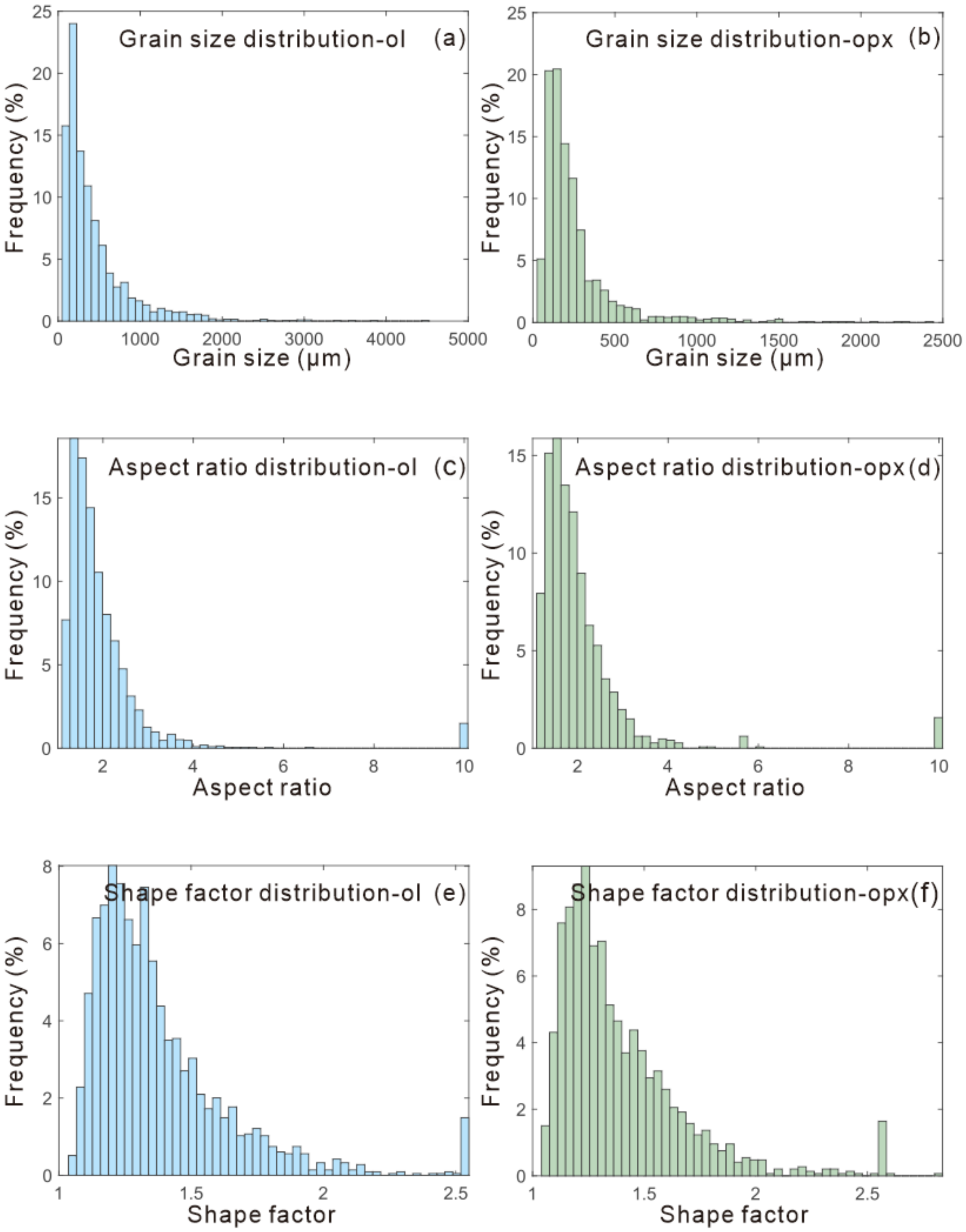

Figure S1. Distributions of (a and b) grain size, (c and d) aspect ratio, and (e and f) shape factor of olivine (left column) and orthopyroxene (right column) grains.

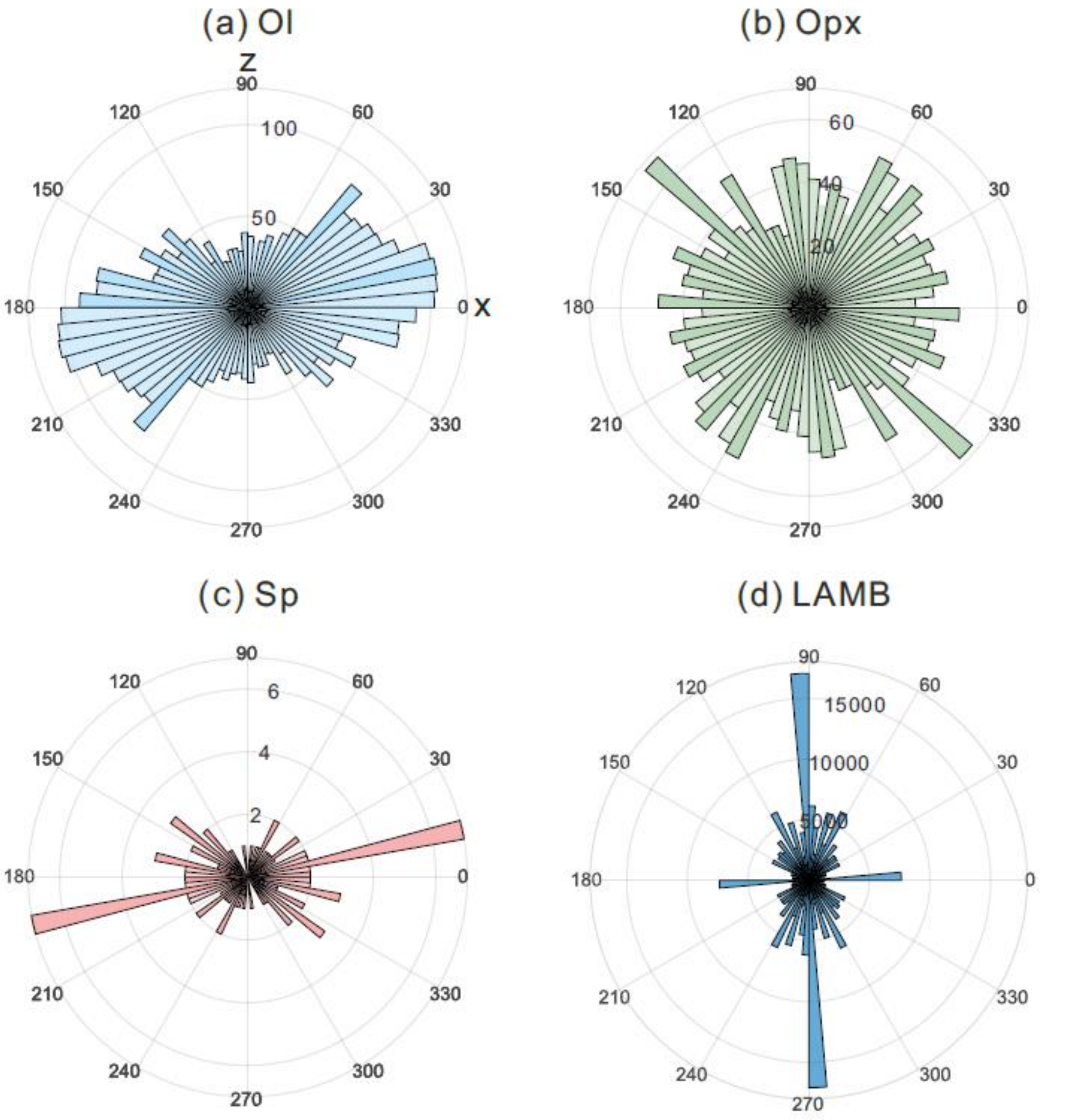

Figure S2. Rose diagrams showing the orientations of the long axes of (a) olivine, (b) orthopyroxene, and (c) spinel grains, and the directions of (d) LAMBs in olivine. The horizontal axis (X-axis) is the lineation direction, and the vertical axis (Z-axis) is the foliation-normal direction.

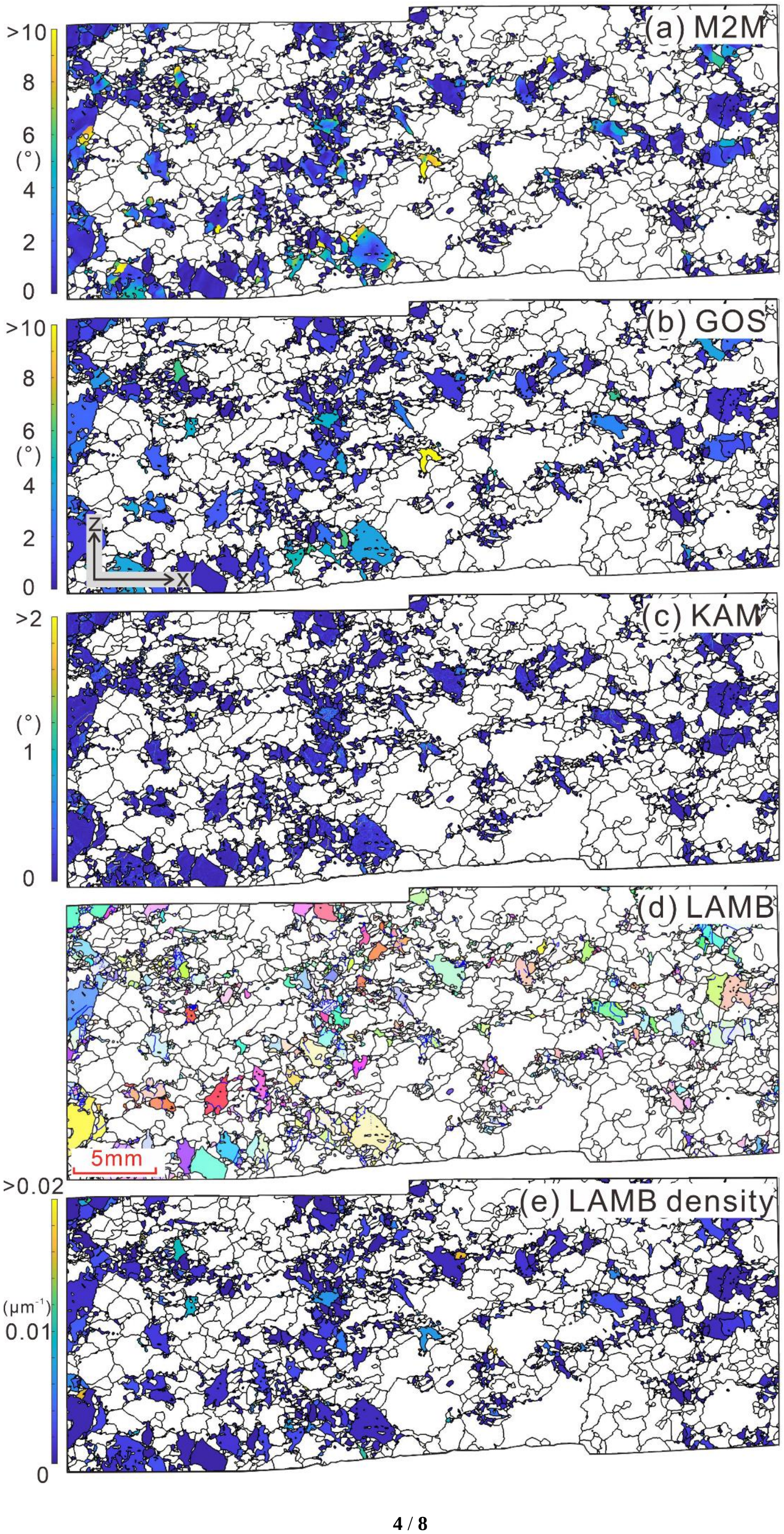

(a) M2M
>10
8
6
(°)
4
2
0
(b) GOS
>10
8
6
(°)
4
2
0
Z
X
(c) KAM
>2
(°)
1
0
(d) LAMB
5mm
(e) LAMB density
>0.02
(μm⁻¹)
0.01
0

Figure S3. Microstructures of orthopyroxene in the studied harzburgite sample. (a) M2M, (b) GOS and (c) KAM of orthopyroxene grains (colored). (d) Distribution of LAMB (blue curves) and (e) LAMB density in orthopyroxene grains. EBSD data were collected using the step size of 15 μm.

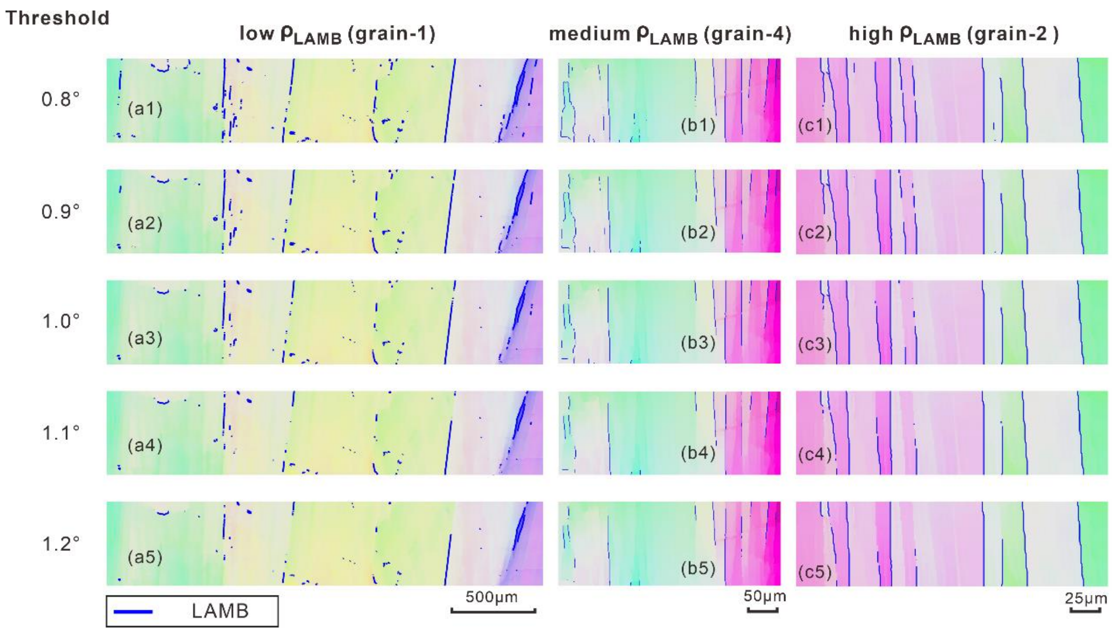

Figure S4. LAMBs (blue lines) vary with the misorientation threshold from 0.8° to 1.2°. (a1-a5) Grain-1, (b1-b5) grain-4 and (c1-c5) grain-2 are representative of the low, medium, and high LAMB density grains, respectively. All grains are color-coded with their orientations.

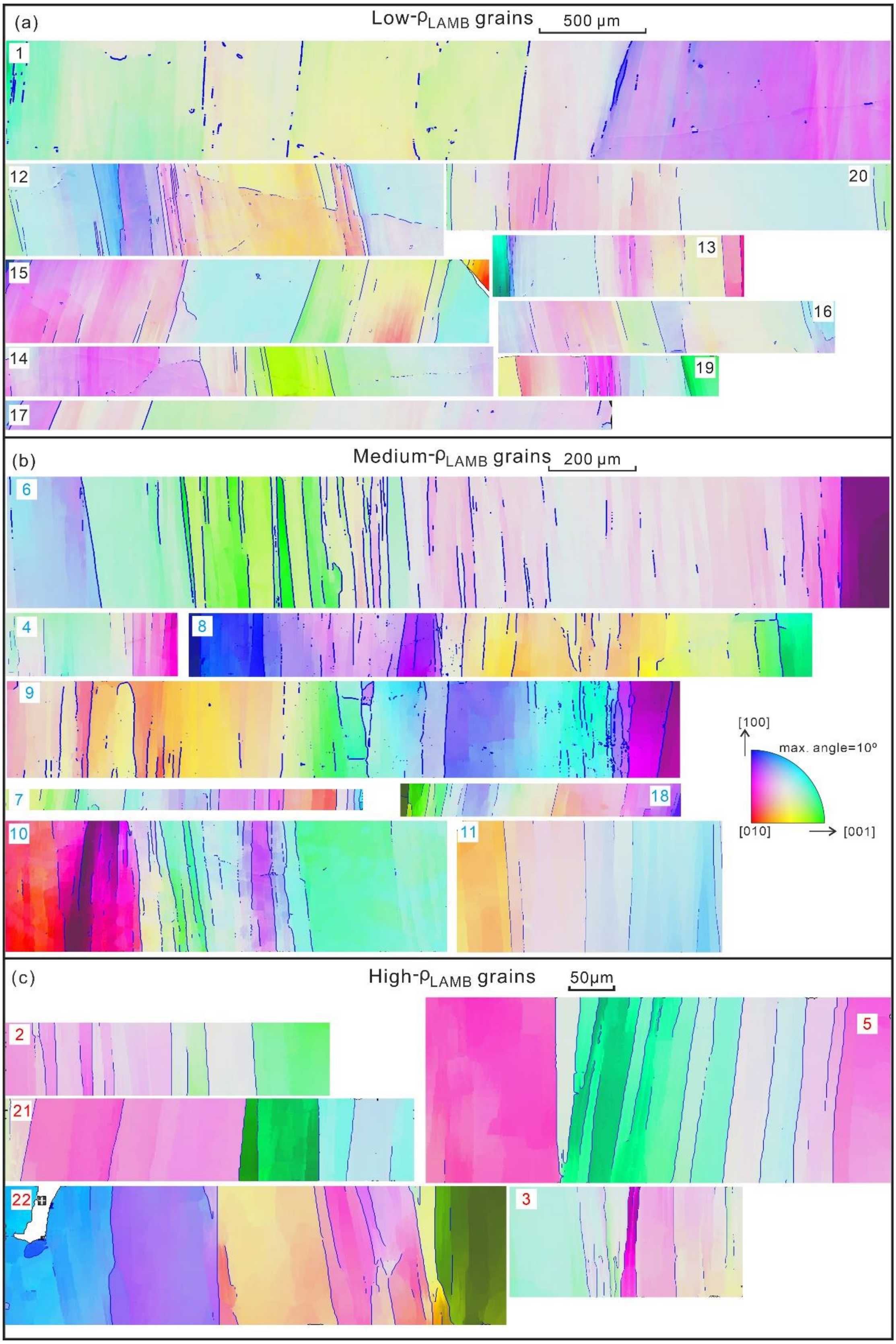
(a)
Low-ρLAMB grains
500 μm
1
12
20
13
15
16
14
19
17
(b)
Medium-ρLAMB grains
200 μm
6
4
8
9
7
18
10
11
[100]
max. angle=10º
[010]
[001]
(c)
High-ρLAMB grains
50μm
2
5
21
22
3

Figure S5. LAMBs (blue lines) in the 22 analyzed olivine grains for the step size of 1 μm. Olivines are categorized into (a) low, (b) medium, and (c) high LAMB density grains, corresponding to those shown in Fig. 6. All grains are color-coded with their orientations. Owing to the large difference in mapping area or grain size, three different scale bars are used here.
